# Design, Synthesis, *In Silico* ADME, Toxicity Prediction and Molecular Docking Studies of Benzimidazole-Oxadiazole Derivatives for α-Glucosidase and Aldose Reductase Pathways as Potent Anti-Diabetic Agents

**DOI:** 10.3390/molecules31152635

**Published:** 2026-07-29

**Authors:** Mesut Işık, Abdüllatif Karakaya, Ulviye Acar Çevik, Adem Necip, Hatice Esra Duran, Bilge Çiftçi, Yusuf Özkay, Zafer Asım Kaplancıklı, Şükrü Beydemir

**Affiliations:** 1 Department of Bioengineering, Faculty of Engineering, Bilecik Şeyh Edebali University, 11230 Bilecik, Turkey; mesut.isik@bilecik.edu.tr; 2 Department of Pharmaceutical Chemistry, Faculty of Pharmacy, Zonguldak Bulent Ecevit University, 67600 Zonguldak, Turkey; a.karakaya@beun.edu.tr; 3 Department of Pharmaceutical Chemistry, Graduate School, Anadolu University, 26470 Eskisehir, Turkey; 4 Department of Pharmaceutical Chemistry, Faculty of Pharmacy, Anadolu University, 26470 Eskisehir, Turkey; yozkay@anadolu.edu.tr (Y.Ö.); zakaplan@anadolu.edu.tr (Z.A.K.); 5 Department of Pharmacy Services, Vocational School of Health Services, Harran University, 63300 Şanlıurfa, Turkey; ademnecip@harran.edu.tr; 6 Department of Medical Biochemistry, Faculty of Medicine, Kafkas University, 36100 Kars, Turkey; hatice.duran@kafkas.edu.tr; 7 Vocational School of Health Services, Bilecik Şeyh Edebali University, 11230 Bilecik, Turkey; bilge.ciftci@bilecik.edu.tr; 8 The Rectorate of Bilecik Şeyh Edebali University, 11230 Bilecik, Turkey; 9 Department of Biochemistry, Faculty of Pharmacy, Anadolu Universty, 26470 Eskisehir, Turkey; sukrubeydemir@anadolu.edu.tr

**Keywords:** benzimidazole, oxadiazole, α-glucosidase, aldose reductase, molecular docking

## Abstract

In this study, due to the side effect profiles and low efficacy of currently used inhibitors, novel benzimidazole-oxadiazole derivatives (**6a**–**6e**, **7a**–**7e**) were synthesized as dual inhibitors of α-GLY and AR. Their structures were elucidated using ^13^C-NMR and ^1^H-NMR techniques. Their binding properties were investigated by molecular docking studies, and their ADME properties were screened *in silico*. AR and α-GLY inhibitory effects of the synthesized compounds were examined. The compounds were observed to exhibit partially similar inhibitory effects to the reference drug epalrestate (*IC*_50_: 0.78 nM; *K*_I_: 0.74 ± 0.0 nM) on AR inhibition. Among them, compounds **6a**, **6b**, and **6c** showed the highest activity with *K*_I_ values of 8.6 ± 0.4, 3.5 ± 0.3 and 6.3 ± 0.5 nM, respectively. Compounds **6a** and **7e** were found to have higher inhibitory activity against the α-GLY enzyme than the reference drug Acarbose (*IC*_50_: 128.4 µM; *K*_I_: 96.2 ± 5.7 µM) with *K*_I_ values of 5.8 ± 0.4 and 7.9 ± 0.8 µM, respectively. Overall, the newly synthesized compounds demonstrated pronounced AR inhibitory activity and notable α-GLY inhibition. Nevertheless, further pharmacological and toxicity evaluations are required to confirm their therapeutic potential. Among the tested molecules, compounds **6a** and **7e** may therefore be considered potential candidates for further investigation as anti-diabetic agents.

## 1. Introduction

Diabetes mellitus (DM) is a chronic metabolic disease characterized by hyperglycemia and glucose intolerance leading to complications such as nephropathy, retinopathy, neuropathy, and cardiovascular disease. DM is associated with impaired insulin secretion, insulin resistance and, in later stages, β-cell dysfunction [1]. It is one of the oldest and most common diseases worldwide [2]. The World Health Organization (WHO) estimates that diabetes will affect more than 19% of the world’s adult population by 2030. Diabetes mellitus is a major public health problem around the world because it is so common and deadly, and the treatment and follow-up costs are so high. Diabetes mellitus is particularly prevalent in developing countries due to the high number of unreported cases [3].

**Scheme 1:**
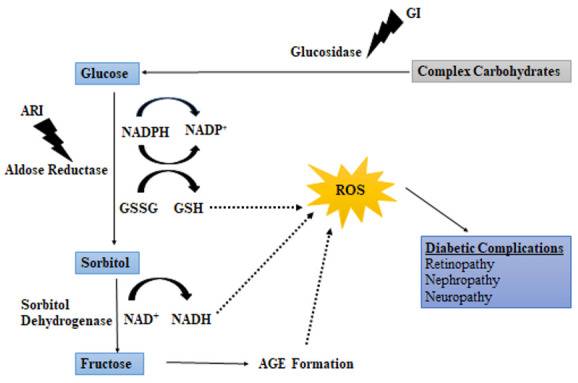
The figure shows the process of conversion of complex carbohydrates to glucose and the illustration of the polyol treatment of glucose. Digestive processes: Complex carbohydrates are broken down by the enzyme α-glucosidase and converted into glucose. After absorption, the glucose taken into the cell is reduced to sorbitol by the AR enzyme with the help of the cofactor NADPH. Sorbitol is converted to a limited extent into fructose by the enzyme sorbitol dehydrogenase, which is expressed in small amounts. Excessive accumulation of sorbitol leads to intracellular osmotic stress. In addition, NADPH consumption contributes to oxidative stress by increasing GSH degradation and ROS. AGE accumulates at the end of these fragments, causing a number of diabetic diseases such as retinopathy, nephropathy and neuropathy [13,14]. The use of inhibitors reduces both postprandial hyperglycemia by limiting the work of glucose and the breakdown of glucose by stressing the sorbitol pathway (AR: aldose reductase, ROS: reactive oxygen species, AGE: advanced glycation end products, ARI: aldose reductase inhibitor, GI: glucosidase inhibitor (α-glucosidase inhibitor)).

DM is clinically divided into two main groups, Type 1 and Type 2. However, there is also gestational DM, which occurs during pregnancy, and genetic or endocrine-related forms. Type 1 DM is characterized by hyperglycemia that develops due to an acute or chronic insulin deficiency in the plasma. In type 2 DM, the β-cells in the islets of Langerhans of the pancreas exhibit increased sensitivity to plasma glucose, resulting in high levels of insulin being released into the systemic circulation. The resulting hyperinsulinemia is a consequence of efforts to control hyperglycemia, and it further impairs β-cell function, causing disease progression [4]. Hyperglycemia due to uncontrolled DM triggers abnormal metabolic processes that play a role in the pathogenesis of diabetic complications by affecting the expression and activity of various metabolic enzymes. Therefore, these enzymes are considered potential therapeutic targets in the treatment of diabetes [5]. One of the most important strategies to control DM, preventing the digestion of dietary carbohydrates, can be achieved by inhibiting the enzyme α-glucosidase (α-GLY) in the small intestine [6]. α-GLY catalyzes the hydrolysis of long-chain carbohydrates into monosaccharides. Inhibition of this enzyme delays the digestion of carbohydrates and provides late carbohydrate absorption. Thus, postprandial hyperglycemia is prevented. In this way, nutrition-based glycemic control is pharmacologically optimized [7]. In this context, inhibition of α-GLY has been shown to be an important therapeutic target that has the potential to lower blood glucose levels by reducing carbohydrate digestion. However, α-GLY inhibitors mainly regulate blood glucose levels without directly affecting insulin secretion. Therefore, these agents are considered first-line oral antidiabetic agents and are used as monotherapy in mild diabetes cases and as combination treatment with insulin or other antidiabetic agents in more severe diabetes cases [8,9]. The three main α-GLY inhibitors currently available for clinical use, acarbose, voglibose, and miglitol, may affect patient compliance due to their limited efficacy and gastrointestinal side effects such as diarrhea, abdominal discomfort, bloating, and flatulence. For these reasons, there is a need to discover new inhibitor compounds that are more effective and have fewer side effects [9]. Aldose reductase (AR) is a key enzyme of the polyol pathway and is physiologically present in many tissues of the body, especially in the eye. This enzyme reduces glucose to sorbitol in the presence of the cofactor NADPH, while the enzyme sorbitol dehydrogenase oxidizes sorbitol to fructose in the presence of NAD^+^ [10,11]. However, the expression of sorbitol dehydrogenase is quite low in many tissues, so the complete conversion of sorbitol into fructose does not take place. As a result, sorbitol accumulates in the cell. Sorbitol is an osmotically active compound, and its excessive accumulation in tissues plays a role in the development of cataracts, glaucoma and other diabetes-related microvascular complications. Therefore, the development of aldose reductase inhibitors (ARIs) is an important strategy for the prevention and treatment of diabetic complications. Although many aldose reductase inhibitors have been identified to date, they have not received FDA approval for reasons such as adverse side effects, low bioavailability, poor pharmacokinetic properties, and insufficient therapeutic efficacy. Known inhibitors include compounds such as epalrestat, tolrestat, zenarestat, ponalrestat, sorbinil, fidarestat, imirestat, ranirestat, and minalrestat [6]. Of these agents, epalrestat is currently the only aldose reductase inhibitor approved for clinical use in Japan, China, and India. In this context, new inhibitory compounds targeting aldose reductase are needed [12]. Consequently, the identification of α-GLY and AR inhibitors holds promise for the development of effective therapeutic strategies for diabetes and its associated complications (Scheme 1).

Heterocyclic ring systems containing nitrogen and oxygen atoms are among the favorite topics in medicinal fields. Among these, benzimidazole is an important group that is similar to naturally occurring nucleotide and purine derivatives in the body. Its ability to form pi-pi binding in targeted proteins, its capacity to be both a donor and acceptor for hydrogen bonding, and its coordination with metals make it a focal point in medicinal chemistry studies [15]. The oxadiazole portions of benzimidazole-oxadiazole derivative compounds synthesized as hybrids are considered bioisosteres, especially for 1,3,4-oxadiazole amides and esters, and are used in hybrid drug synthesis. It plays an important role in drug design because it increases affinity through strong hydrogen bonds as well as improves metabolic stability [16]. Due to the multifaceted and complex etiology of diabetes mellitus, single-drug designs are sometimes insufficient. In these cases, drug designs containing multiple pharmacophore groups, such as benzimidazole-oxadiazole hybrids, are used [17]. A hybrid approach combining benzimidazole and oxadiazole rings has enabled the creation of dual-acting designs targeting α-GLY and AR. In this hybrid design, benzimidazole facilitates the main interactions with the target receptor while oxadiazole is responsible for increasing affinity and stability [18]. Due to their low side effects and high pharmacological effects, benzimidazole-oxadiazole derivative novel compounds are heterocyclic structures that attract attention in terms of their anti-diabetic potential. These derivatives are emerging as potential antidiabetic agents, particularly by inhibiting the enzymes α-GLY and AR. The benzimidazole-oxadiazole structure, with its pharmacophore properties, exhibits high affinity for these enzymes. Furthermore, their bioavailability can be increased through various modifications [19,20]. Computational approaches have become valuable tools in modern drug discovery by providing insights into molecular interactions and structure–activity relationships. In this context, integrating in silico analyses with experimental studies can support the identification of promising bioactive compounds. These collective findings reinforce the significance of hybrid scaffold design, integrating computational and experimental methodologies, in advancing next-generation therapeutic agents [21,22]. This study reports on the design and synthesis of novel benzimidazole-oxadiazole derivatives that target the inhibition of α-GLY and AR enzymes, as well as the in silico and in vitro bioactivity analysis of these compounds as potential anti-diabetic agents. We evaluated the binding affinities of the synthesized compounds toward the α-GLY and AR proteins using molecular docking studies. Additionally, we predicted the potential effects and pharmacokinetic properties of these molecules on human metabolism using ADME (absorption, distribution, metabolism, elimination) calculations.

## 2. Results and Discussion

### 2.1. Chemistry

**Scheme 2:**
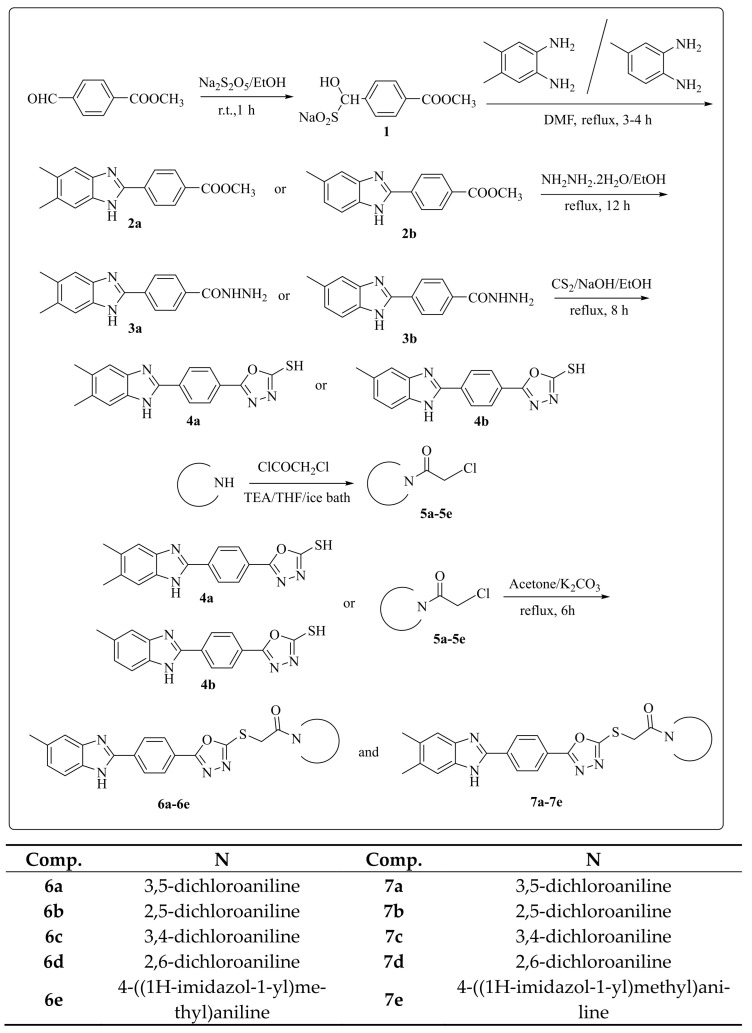
General procedure for the synthesis of the final compounds **6a**–**6e** and **7a**–**7e**.

The synthesis of the targeted benzimidazole-oxadiazole derivatives (**6a**–**6e**, **7a**–**7e**) was carried out as shown in Scheme 2. In the first step, the salt of the aldehyde is obtained by means of sodium metabisulfite in ethanol. The formed salt is reacted with 4-methyl-*o*-phenylenediamine and 4,5-dimethyl-*o*-phenylenediamine in DMF to obtain compounds (**2a**, **2b**). Hydrazide derivative compounds (**3a**, **3b**) are obtained by reacting (**2a**, **2b**) and hydrazine hydrate in ethanol. The mixture of hydrazide derivative compounds (**3a**, **3b**) is reacted with CS_2_ in the presence of basic catalyst NaOH in ethanol to obtain compounds (**4a**, **4b**) containing benzimidazole and oxadiazole rings. To obtain acetylated aniline (**5a**, **5e**) derivatives, chloroacetyl chloride was reacted with aniline derivatives in THF in the presence of TEA. To synthesize the target compounds (**6a**–**6e**, **7a**, **7e**), compounds (**4a**, **4b**) and (**5a**, **5e**) were reacted with acetone and potassium.

### 2.2. Biological Activity

The inhibitory characteristics of the benzimidazole oxadiazole derivatives **6a**–**6e** and **7a**–**7e** against AR and α-GLY enzymes are examined in this study. The main goals of the study were to determine the possible effectiveness of these derivatives and to offer suggestions for creating new therapeutic agents that block enzymes linked to diabetes. Table 1 presents the results and inhibition data for benzimidazole oxadiazole derivatives **6a**–**6e**, **7a**–**7e**, and reference compounds as positive controls.

The benzimidazole-oxadiazole derivatives **6a**–**6e**, **7a**–**7e** were evaluated for their inhibitory effects on AR and α-GLY, enzymes associated with DM. The inhibitory potential of these compounds against the AR enzyme was measured in the range of IC_50_ values of 4.93–19.22 nM and *K*_I_ values of 3.5 ± 0.3–27.7 ± 2.0 nM. Although all compounds showed lower inhibitory potential on the AR enzyme compared to the reference inhibitor epalrestat (IC_50_: 0.78 nM; *K*_I_: 0.74 ± 0.0 nM), they can be said to exhibit significant inhibitory potential at the nanomolar level. The *K*_I_ values provide important information about the affinity and selectivity of inhibitors towards the target enzyme. As shown in Table 1, all synthesized derivatives have the highest selectivity over AR. According to these results, the compounds **6a**, **6b**, and **6c** were found to be the strongest AR inhibitors with *K*_I_ values of 8.6 ± 0.4, 3.5 ± 0.3 and 6.3 ± 0.5 nM, respectively. In contrast, although compound **7b** has a weaker inhibitory effect compared to other derivatives, it has a higher inhibitory effect than the reference inhibitor epalrestat. Because of their superior inhibitory effects on the AR enzyme when compared to the reference inhibitor epalrestat, the benzimidazole-oxadiazole derivatives synthesized in the study are gaining attention as possible therapeutic agents. Kinetic studies on the α-GLY enzyme were used to assess the compounds’ antidiabetic potential; IC_50_ values ranged from 5.8 µM to more than 400 µM and *K*_I_ values ranged from 5.9 ± 0.4 µM to >400 µM. According to the data obtained, compounds **6a**, **7b**–**7e** showed significantly higher inhibitory activity on α-GLY compared to the reference inhibitor acarbose (IC_50_: 128.4 µM; *K*_I_: 96.2 ± 5.7 µM). Of the substances that were tested, compounds **6a** and **7e** stood out as the most potent α-GLY inhibitors with *K*_I_ values of 5.8 ± 0.4 and 7.9 ± 0.8 µM, respectively. These two compounds demonstrated the highest selectivity over both AR and α-GLY enzymes in terms of enzyme kinetics, whereas compounds **6b**–**6e** demonstrated the lowest selectivity towards the α-GLY enzyme. Table 1 summarizes these results. The highest selectivity for AR was observed for compound **6b**, while compounds **6a** and **7e** were the most remarkable compounds in terms of α-GLY. Given these results, the compounds **6a**–**6e**, **7a**–**7e** show significant inhibitory effects on both AR and α-GLY compared to reference inhibitors such as epalrestat and acarbose, highlighting them as potential multitarget antidiabetic candidates. The inhibitory activity of oxadiazole derivatives against various biological targets has been emphasized in many studies in the literature. The inhibitory potential of the benzimidazole-oxadiazole derivatives synthesized in this study, especially against AR and α-GLY enzymes, reveals once again that this structural scaffold is a biologically important pharmacophore. In one study, the inhibitory potential of compounds consisting of 5-substituted 1,3,4-oxadiazole-2-thione derivatives was investigated against α-GLY. These derivatives showed significant inhibitory activity with IC_50_ values in the range of 43.05–78.27 µM compared to the reference drug acarbose (IC_50_: 145.31 µM) [23]. Similarly, in another study, a series of compounds with a 1,3,4-oxadiazole core exhibited IC_50_ values of 0.80–45.10 µM [24]. The 3,4,5-triphenyl-4,5-dihydro-1,2,4-oxadiazole derivatives synthesized by Khosravi and coworkers showed that compounds with chloro, fluoro and methoxy substituents on the aryl ring exhibited significantly higher inhibitory activity than the reference compound (*IC*_50_: 750 ± 12.00 µM) with IC_50_ values of 215.0 ± 3.00 µM, 256.0 ± 3.00 µM and 295.0 ± 4.00 µM, respectively [25]. In another study, coumarin-oxadiazole derivatives (**11a**–**h**) were synthesized and the inhibitory effect of these compounds against AR was investigated. The compounds exhibited IC_50_ values of 0.31–57.5 µM [26]. In the study conducted by Javid et al., oxadiazole-sulfonamide hybrids showed a significant inhibitory effect compared to the reference standard sorbinil (IC_50_: 3.14 µM) with IC_50_ values between 3.06 and 30.6 µM [27]. In this context, all of the benzimidazole oxadiazole derivatives synthesized in this study exhibited higher inhibitory activity against the AR enzyme than the reference inhibitor epalrestat and the oxadiazole derivatives reported in the literature, with an inhibitory potency ranging from 3.523 ± 0.256–27.734 ± 2.023 nM. In a similar vein, all compounds except for compounds **6b**–**6e** exhibited a more potent effect on α-GLY than many oxadiazole derivatives reported in the extant literature. Notably, a significant proportion of the compounds **7a**–**7e** demonstrated a potency level that surpassed not only acarbose but also the majority of the compounds documented in the extant literature.

In summary, the newly synthesized benzimidazole-oxadiazole derivatives possess potent inhibitory profiles against both AR and α-GLY enzymes, positioning them as potential candidate molecules for pharmaceutical development. Further pharmacodynamic and pharmacokinetic evaluations may provide additional insights and support for these molecules.

### 2.3. Molecular Docking Analysis

In this study, the inhibitory potentials of the synthesized compounds **6a**–**6e**, **7a**–**7e** against AR and α-GLY enzymes were comprehensively evaluated using both experimental (enzyme inhibition tests) and computational (molecular docking) methods. The findings clearly demonstrate the decisive role of individual molecular properties in enzymatic activity, as well as intergroup differences, and reveal that some of the synthesized compounds exhibit superior inhibitory activity compared to existing positive control drugs. Molecular docking is a computational modeling approach used to investigate intermolecular interactions by predicting the binding orientation, mechanism, and affinity between a ligand and a biological target. In recent years, this technique has gained increasing importance as a supportive tool in rational drug design. Enabling the evaluation of ligand-protein interactions at the molecular scale, molecular docking contributes significantly to the identification and optimization of potential drug candidates. This method is widely applied in drug discovery and biotechnology research to elucidate the structural and functional behavior of biomolecular systems. Docking studies provide insights into how small molecules interact with the active sites of target proteins and enable the estimation of binding energies associated with these interactions. Compounds exhibiting stronger biological potential generally show more stable interactions, which is reflected in lower docking score values [28,29]. In this study, the binding performance of the synthesized compounds was evaluated by comparing them with reference molecules. The estimated binding scores obtained from ligand-protein interaction analyses are summarized in Table 2.

In this study, the docking affinities of synthesized compounds **6a**–**6e**, **7a**–**7e** toward two key enzymes involved in glucose metabolism as AR (PDB ID: 4JIR) and α-GLY (PDB ID: 3WY1) were evaluated. The results were compared with standard reference inhibitors, epalrestat and acarbose, which are clinically used AR and α-GLY inhibitors, respectively. All the tested compounds exhibited considerably stronger binding affinities toward AR than epalrestat, with docking scores ranging from −11.2 to −11.7 kcal/mol, while epalrestat demonstrated a much lower docking score of −7.3 kcal/mol. Among these, compound **7c** showed the most favorable binding with a score of −11.7 kcal/mol, followed closely by compounds **6a**, **6c**, and **7e**, all of which scored −11.5 kcal/mol. These results strongly suggest that the newly synthesized compounds may possess potent AR inhibitory properties, surpassing the standard drug in binding affinity. Similarly, for α-GLY inhibition, most compounds outperformed acarbose, which exhibited a relatively weak docking score of −5.0 kcal/mol. In particular, compound **7b** (−9.6 kcal/mol), compound **7e** (−9.5 kcal/mol), and compound **6e** (−9.4 kcal/mol) displayed the highest binding affinities.

In experimental evaluations of the AR enzyme, compounds such as compounds **6b** (*K*_I_: 3.5 nM), **6c** (6.3 nM), and **7c** (7.4 nM) showed a significantly stronger inhibitory effect than the reference inhibitor epalrestat (*K*_I_: 0.74 ± 0.0 nM). Additionally, the docking scores of these compounds ranged from −11.4 to −11.7 kcal/mol, significantly higher than epalrestat’s docking score (−7.3 kcal/mol). These data indicate that the compounds in question are strong candidates as inhibitors at both the theoretical and experimental levels.

**Figure 1:**
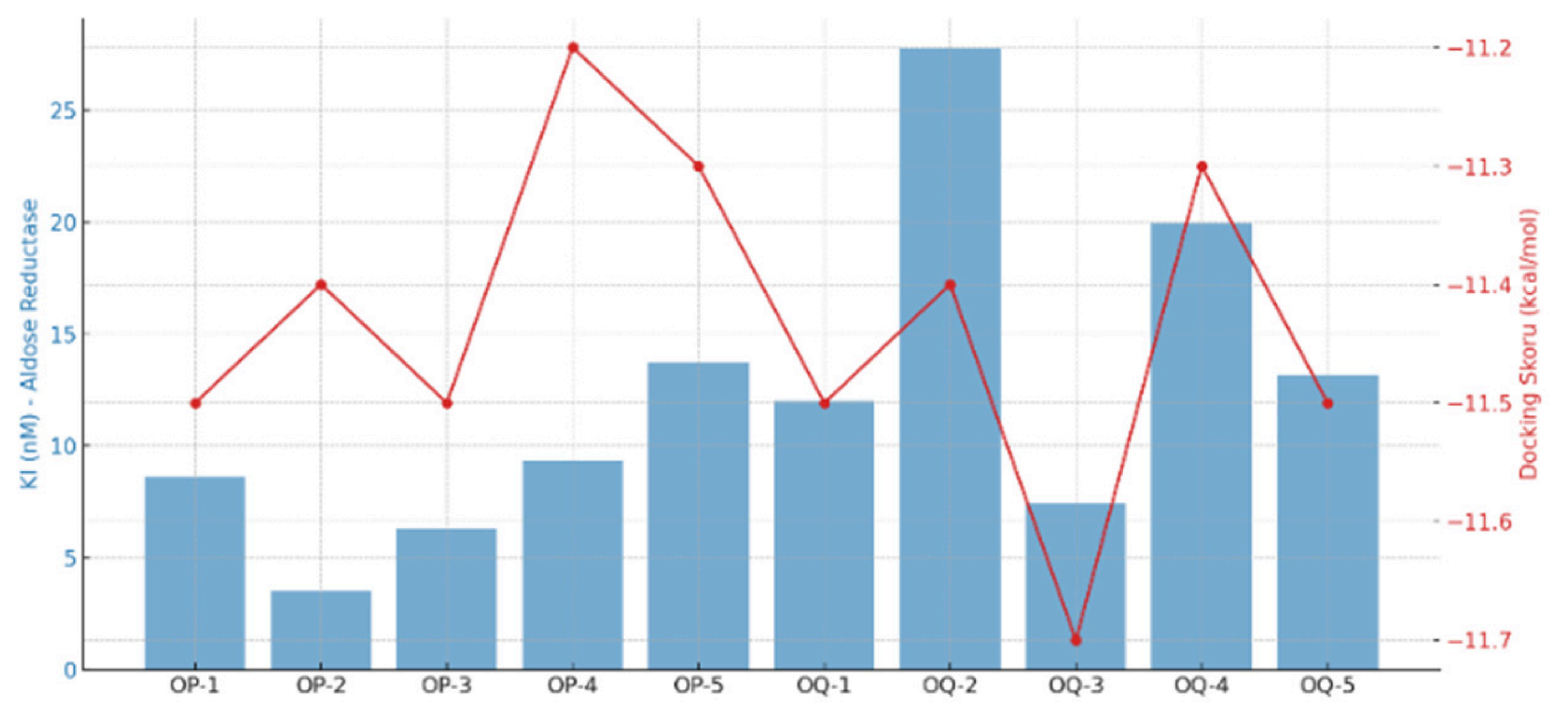
Effects of the compounds **6a**–**6e**, **7a**–**7e** on aldose reductase.

Figure 1 shows a comparative analysis of the effects of 10 newly synthesized molecules on the AR enzyme, both *in vitro* (*K*_I_ values) and *in silico* (docking scores). The blue columns represent the experimental inhibitory effects of the molecules as *K*_I_ (nM). The red line indicates the docking scores (kcal/mol). More negative values indicate stronger binding.

In studies conducted on the α-GLY enzyme, the compounds **6a** (*K*_I_: 5.9 µM) and **7e** (*K*_I_: 7.9 µM) were found to be significantly more effective than the reference inhibitor acarbose (*K*_I_: 96.2 ± 5.72 µM). Furthermore, considering that the docking score of acarbose is quite low at −5.0 kcal/mol, it has been demonstrated that the new compounds (−9.5 to −9.6 kcal/mol) are also superior in terms of their theoretical binding capacity to this enzyme.

**Figure 2:**
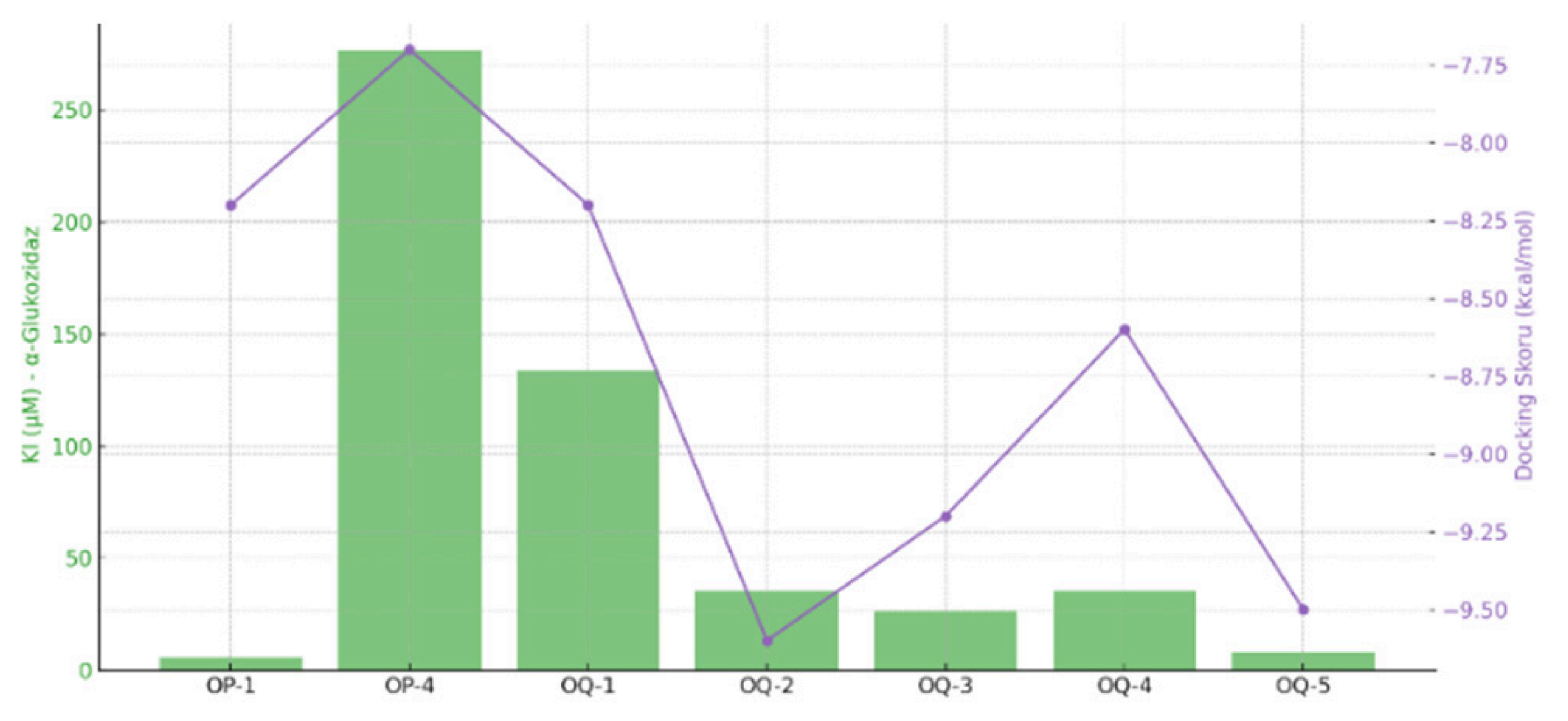
Effects of the compounds **6a**–**6e**, **7a**–**7e** on α-glucosidase.

**Figure 3:**
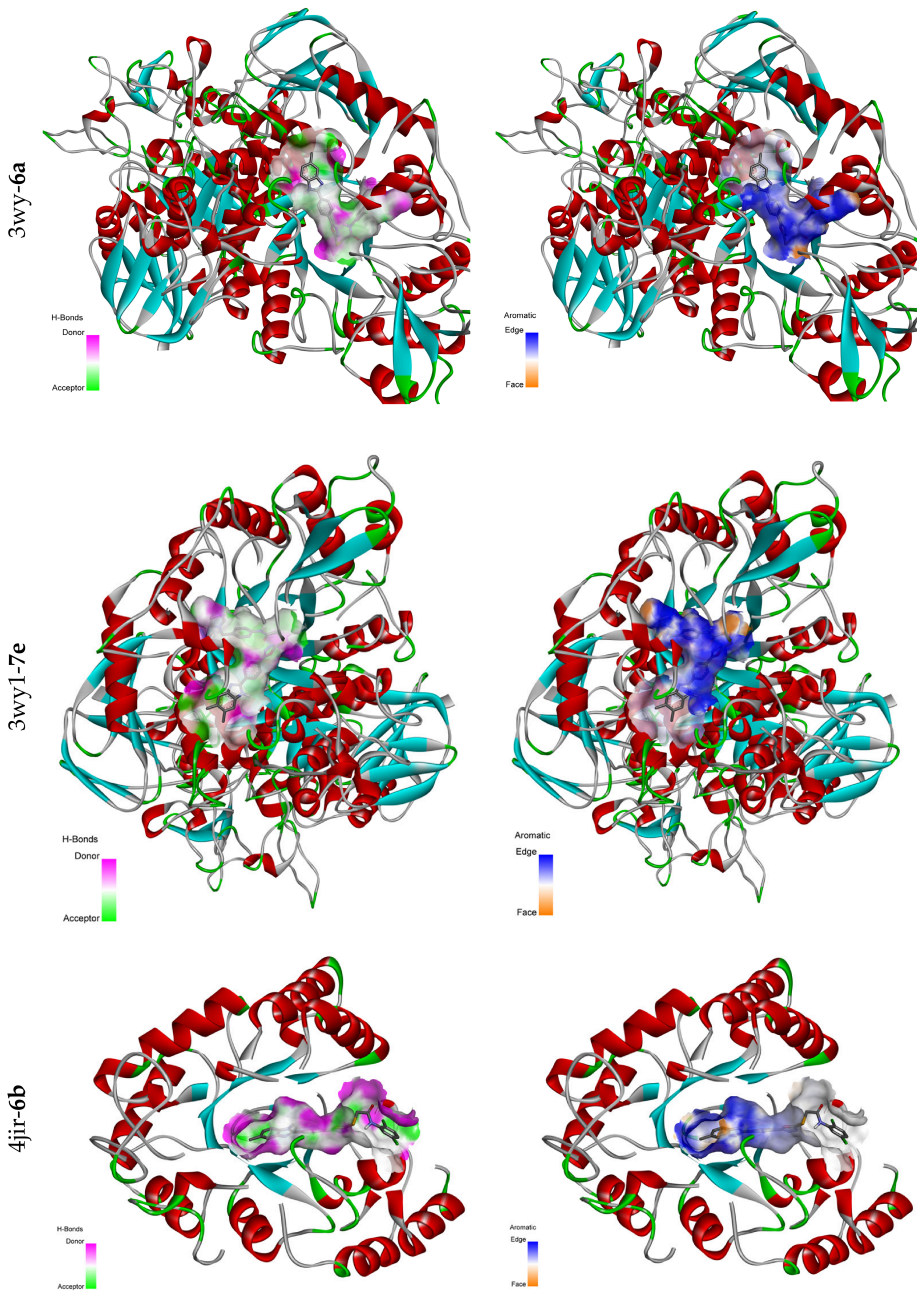

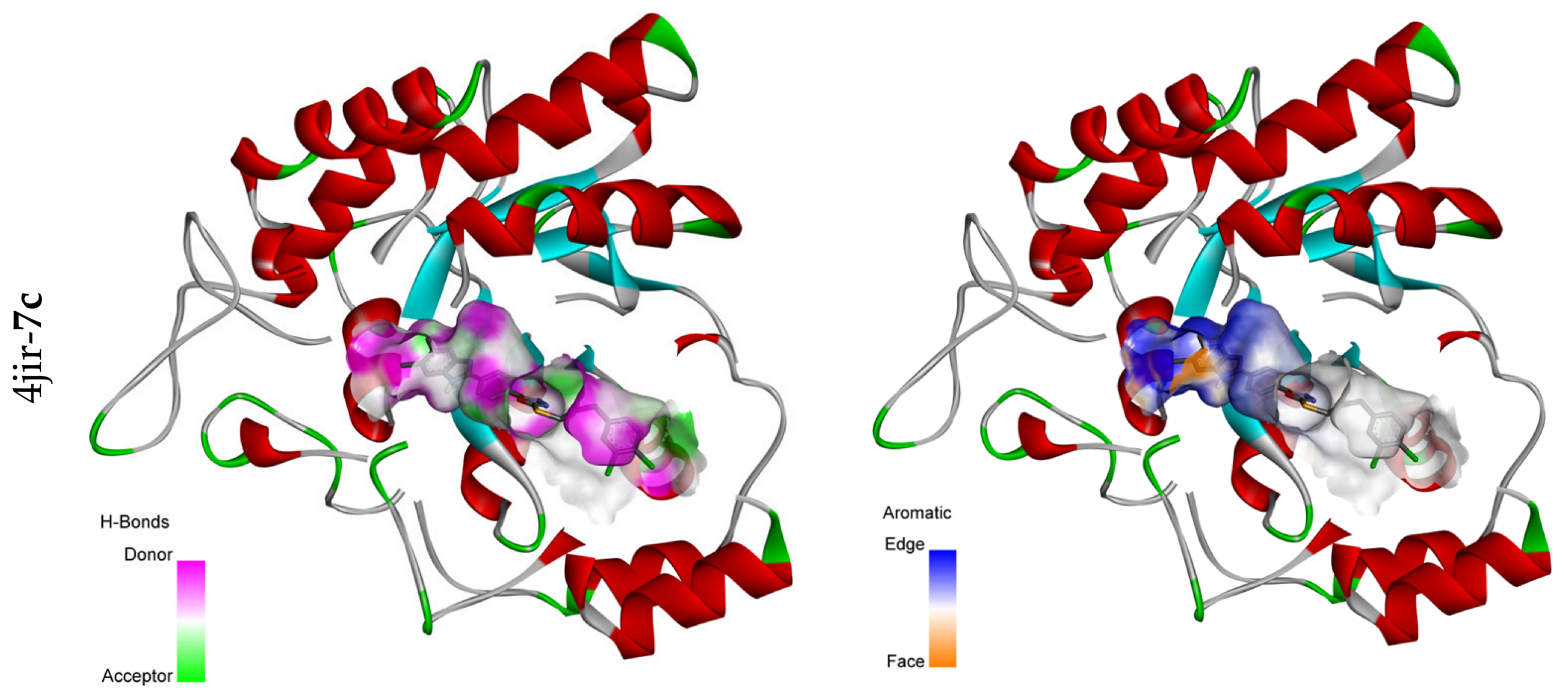
Protein-ligand interaction 2D represented by 3WY1 and 4JIR. Binding mode and visual interaction (H-bonds, Aromatic) of designed **6a**, **7e**, **6b**, and **7c** against 3WY1 and 4JİR.

**Figure 4:**
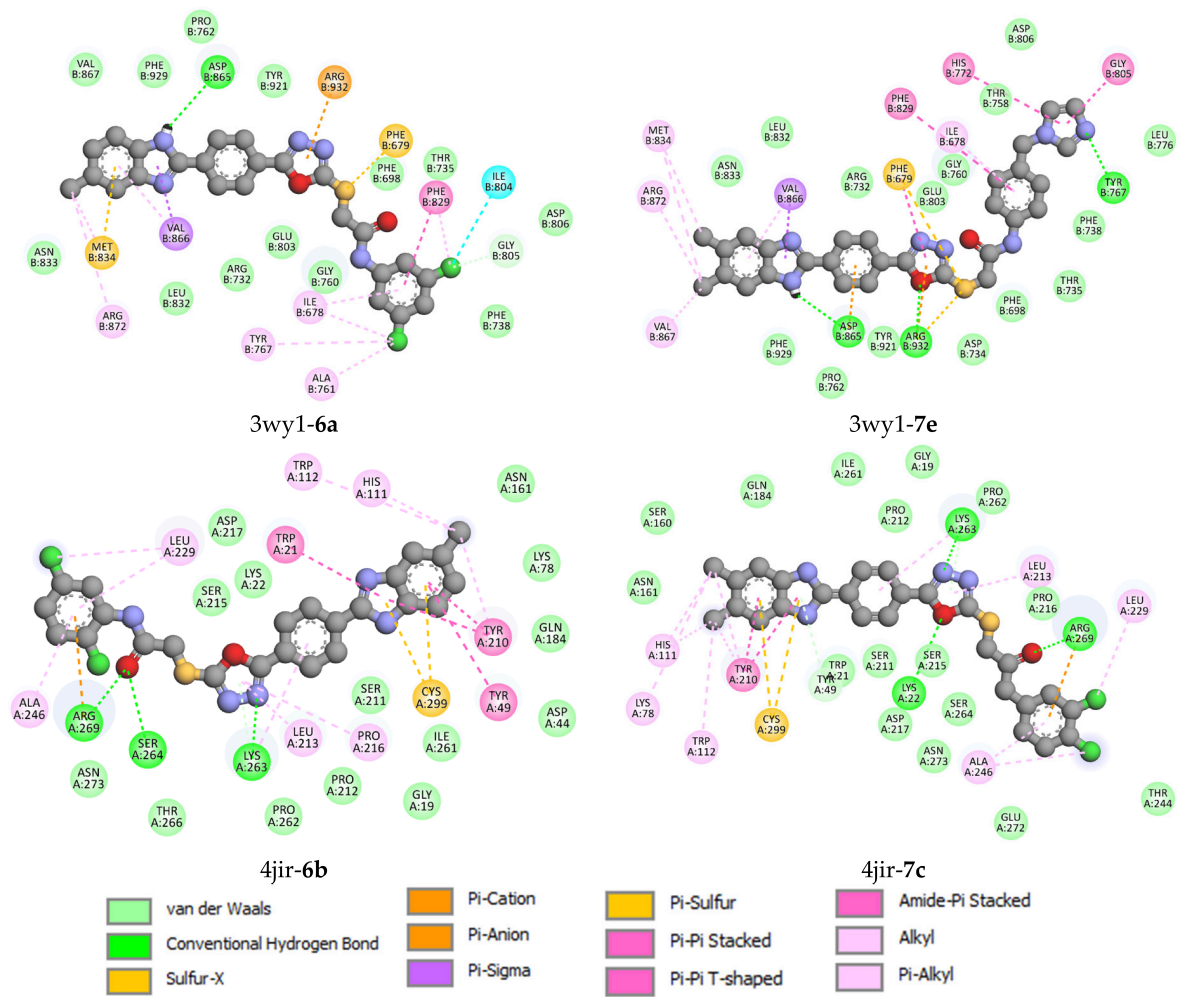
Protein-ligand interaction 3D represented by 3WY1 and 4JIR. Binding mode and visual interaction (H-bonds, Aromatic) of designed compounds **6a**, **7e**, **6b**, and **7c** against 3WY1 and 4JİR.

Figure 2 shows the effects of newly synthesized compounds on the α-GLY enzyme. The green columns represent the in vitro inhibitory effects of the compounds as *K*_I_ (nM). The purple line indicates the in silico docking scores (kcal/mol). The compounds **6a** and **7e** exhibit high inhibitory activity with the lowest *K*_I_ values. The compounds **7b** and **7e** have the strongest docking scores (−9.6 and −9.5 kcal/mol). The docking score values of the compounds binding to AR (4JIR) and α-GLY (3WY1) receptors vary from −11.2 to 11.7 and −7.3 to −9.6 kcal/mol, respectively. When the in vitro and in silico findings of the compounds in Table 2, Figure 3 and Figure 4 are evaluated, it is understood that the compounds have a strong tendency to bind to active sites. Although both groups exhibit strong inhibitory properties, the compounds **7a**–**7e** generally perform better, especially in terms of docking scores. The compounds **7c**, **7e**, and **7b** have shown remarkable results on both AR and α-GLY enzymes. However, compounds **7b**, **7c** are also quite potent in terms of *K*_I_ values and stand out as the potential candidates within the compounds **6a**–**6e**. These differences are likely due to variations in molecular structure and their compatibility with enzyme active sites. The functional groups in the compounds **7a**–**7e** may support this result by providing higher binding energies through hydrogen bonds and hydrophobic interactions. The compounds **6a**–**6c**, **7c**, and **7e** are structures with the highest potential based on both experimental and computational data. These compounds are considered to be strong biological agents that could be developed as alternatives to existing drugs. Additionally, these structures could be integrated into pharmaceutical development processes through further optimization studies based on molecular structure–activity relationships.

Molecular docking analysis revealed several hydrophobic interactions between the compound **6a** and key residues within the active site of α-GLY (PDB ID: 3WY1). Notably, hydrophobic contacts were observed with ILE678B (3.58 Å), PHE829B (3.49 and 3.09 Å), ASP865B (3.93 Å), VAL867B (3.64 Å), TYR921B (3.11 Å), and PHE929B (3.87 Å). These interactions, particularly with aromatic residues such as phenylalanine and tyrosine, are indicative of strong π-π stacking and van der Waals forces that may contribute significantly to the stabilization of the ligand within the enzyme’s active pocket. The proximity of these residues suggests that compound **6a** fits well into the hydrophobic cavity of the enzyme, supporting its high binding affinity and potential inhibitory activity.

Docking analysis of the compound **7e** with α-GLY (PDB ID: 3WY1) revealed multiple hydrophobic interactions that contribute to its strong binding affinity. Specifically, interactions were observed with ILE678B (3.66 Å), PHE829B (3.72 and 3.14 Å), ASP865B (3.84 Å), VAL867B (3.35 Å), ARG872B (3.78 Å), and TYR921B (2.98 Å). The close proximity of aromatic residues such as PHE829B and TYR921B suggests significant π-π stacking interactions, while the involvement of aliphatic side chains (ILE and VAL) supports van der Waals stabilization. These hydrophobic contacts indicate that compound **7e** is well accommodated within the enzyme’s hydrophobic pocket, which may underlie its high docking score and potential inhibitory effect on α-GLY activity. Molecular docking analysis demonstrated that the compound **6b** engages in several hydrophobic interactions within the active site of AR (PDB ID: 4JIR). Key residues involved in these interactions include TRP21A (3.37 Å), TYR210A (3.58 and 3.62 Å), LEU229A (3.77 Å), and LYS263A (3.37 Å). The participation of aromatic residues such as tryptophan and tyrosine suggests the presence of stabilizing π-π interactions, while aliphatic and basic residues such as leucine and lysine may contribute additional van der Waals forces. These hydrophobic contacts likely facilitate a stable ligand conformation within the binding pocket, which is consistent with the high docking affinity observed for compound **6b**. Docking simulations revealed that the compound **7e** establishes extensive hydrophobic interactions within the active site of AR (PDB ID: 4JIR). Key interacting residues include TRP21A (3.20 Å), TRP112A (3.62 Å), TYR210A (3.86 and 3.91 Å), LEU213A (3.48 Å), ALA246A (3.62 Å), LYS263A (3.43 Å), and ARG269A (3.59 Å). The involvement of aromatic residues such as tryptophan and tyrosine suggests strong π-π stacking interactions, while contacts with aliphatic and basic residues (LEU, ALA, LYS, ARG) indicate supportive van der Waals forces and potential electrostatic complementarity. These interactions likely contribute to the high binding affinity of compound **7e**, facilitating its stable accommodation within the enzyme’s hydrophobic pocket.

Hydrogen bonding analysis provided further insight into the binding stability of the compounds with their respective target enzymes. For the α-GLY(3WY1)–ligand complexes, compound **6a** formed a single hydrogen bond with ASP865B (H–A: 2.68 Å; D–A: 3.16 Å; angle: 110.64°), whereas compound **7e** formed two hydrogen bonds: one with ASP865B (2.40 Å) and another with ARG932B (3.41 Å), indicating a slightly enhanced interaction profile compared to compound **6a**. In the case of AR (4JIR), compound **6b** exhibited hydrogen bonding with LYS263A (2.54 Å) and ARG269A (2.41 Å), with favorable donor angles of 155.35° and 129.61°, respectively. Notably, compound **7e** formed even stronger hydrogen bonds with the same residues—LYS263A (2.16 Å; angle: 166.84°) and ARG269A (2.53 Å)—suggesting a more stable and energetically favorable interaction within the enzyme’s active site. Overall, these hydrogen bonds, particularly those with shorter H–A distances and larger donor angles, likely contribute significantly to the high binding affinities observed in the docking studies, enhancing the stability and specificity of ligand–target interactions. The molecular docking analysis identified notable π-cation interactions between the aromatic moieties of the ligands and positively charged arginine residues in both target enzymes. In the α-GLY (3WY1) complexes, compound **6a** and compound **7e** formed π-cation interactions with ARG932B at distances of 4.42 Å and 4.24 Å, respectively. Similarly, in the AR (4JIR) complexes, compound **6b** and compound **7e** interacted with ARG269A via π-cation forces at 3.65 Å and 4.05 Å, respectively. These interactions, involving electrostatic attraction between the electron-rich aromatic rings of the ligands and the cationic guanidinium group of arginine, are known to enhance binding stability and specificity. The presence of π-cation interactions further supports the strong binding affinities observed in the docking results and suggests a potential role in the inhibitory activity of the compounds. In the AR (PDB ID: 4JIR) docking complexes, both compounds **6b** and **7e** exhibited π–π stacking interactions with the aromatic side chain of TYR210A. The interaction distances were measured as 3.67 Å for compound **6b** and 3.56 Å for compound **7e**, with respective interplanar angles of 2.31° and 2.33°, indicating nearly parallel (P-type) stacking conformations. These π–π interactions contribute to the stabilization of the ligand within the enzyme’s binding pocket by enhancing van der Waals and electrostatic interactions between aromatic systems. Their presence supports the high binding affinity and stable orientation of both compounds in the active site of AR. In the AR complex with compound **6b** (PDB ID: 4JIR), halogen bonding interactions were observed with ASN273A at distances of 3.39 Å and 3.31 Å. The respective donor angles were 156.42° and 141.49°, while the acceptor angles measured 95.27° and 101.12°. These geometries fall within typical parameters for halogen bonds, indicating a favorable directional interaction between the halogen atoms of compound **6b** and the polar side chain of asparagine. Such halogen bonds may enhance ligand binding specificity and contribute to the overall stability of the ligand-enzyme complex (Table 3).

The pharmacokinetic properties of the newly synthesized compounds **6a**, **6b**, **7c**, and **7e** were evaluated *in silico* to assess their drug-likeness and potential oral bioavailability. The analysis included key physicochemical parameters such as molecular weight, topological polar surface area (TPSA), hydrogen bonding capacity, lipophilicity (logP/logD), solubility (logS), and rule-based assessments (Lipinski, Pfizer, GSK, and Golden Triangle filters), all of which are pivotal in early-stage drug design. All four compounds exhibited molecular weights ranging between 509.05 and 535.18 g/mol. Compounds **6a**, **6b**, and **7c** exceed the classical Lipinski threshold of 500 g/mol, which may negatively impact passive membrane permeability and oral bioavailability. Compound **7e** remained within the acceptable range, presenting a comparatively more favorable profile in this regard. Regarding polarity, all molecules demonstrated TPSA values within the optimal range (<140 Å^2^), with compound **7c** having the lowest (84.67 Å^2^) and compound **7e** the highest (114.52 Å^2^). The relatively moderate TPSA of compound **7c** suggests an increased potential for transcellular absorption, while the higher TPSA of compound **7e** could hinder membrane permeability to some extent. The higher TPSA of compound **7e** may moderately limit membrane permeability, yet this is counterbalanced by its more favorable lipophilicity and solubility profile. Hydrogen bond acceptor (nHA) and donor (nHD) counts for the molecules were within acceptable limits, enhancing their capacity to participate in specific interactions with biological targets. Compound **7e** had the highest number of hydrogen bond acceptors (nHA = 9), indicating a strong potential for polar interactions, which could be beneficial for binding specificity. The logP values of compounds **6a** and **6b** (5.459) and compound **7c** (6.123) suggest high lipophilicity, potentially enhancing membrane permeability but also raising concerns about bioaccumulation and off-target toxicity. In contrast, compound **7e** exhibited a more balanced logP value (3.635), indicating a favorable hydrophilic-lipophilic balance conducive to drug-likeness. Consistent with these findings, only compound **7e** met the Lipinski rule of five criteria, further supporting its potential as a drug candidate. Solubility, assessed via logS, revealed poor aqueous solubility for all compounds, with compound **7c** displaying the lowest solubility (logS = −7.08), which may pose challenges for formulation and absorption. Nevertheless, all compounds passed the Pfizer rule, indicating acceptable solubility and logP parameters from a toxicity risk perspective. All four molecules failed to satisfy the GlaxoSmithKline (GSK) and Golden Triangle rules, which consider an optimal balance between molecular weight, lipophilicity, and TPSA. This suggests a need for structural optimization to improve metabolic stability and pharmacokinetic behavior. Compound **7e**, as the only compound satisfying Lipinski’s Rule of Five and displaying the most favorable overall physicochemical profile within the series, represents the most suitable starting point for future lead optimization efforts (Table 4).

**Figure 5:**
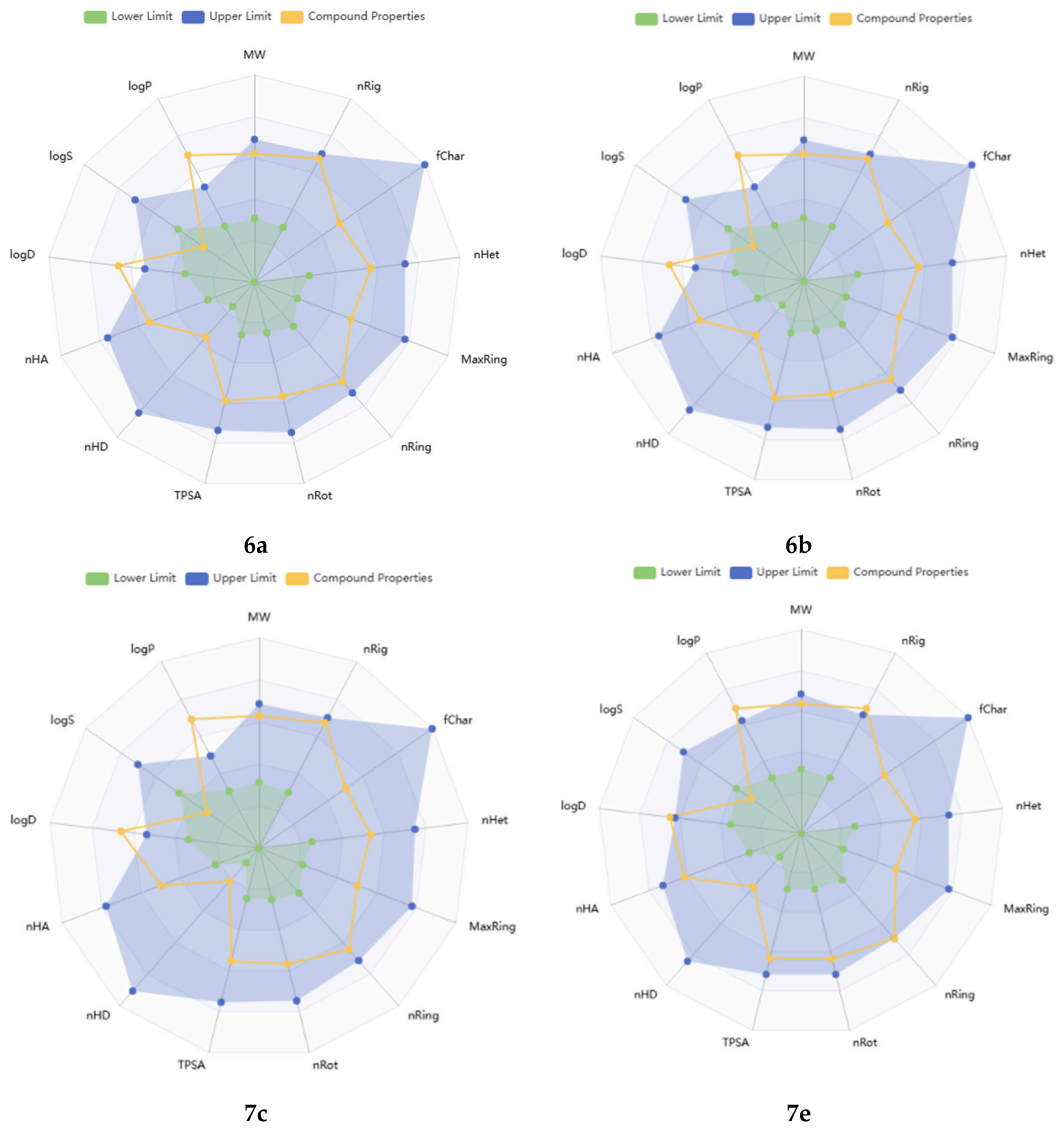
Plot radar graph showing the chemical structure and physicochemical properties of compounds **6a**, **6b**, **7c**, and **7e**.

The chemical structure (compounds **6a**, **6b**, **7c**, and **7e**) and the physicochemical properties of this molecule are illustrated by a radar plot (spider plot). The green line indicates the lower limit of the molecule’s properties; the yellow line indicates the compound’s properties; the blue line indicates the lower limit of the molecule’s properties. Overall, compounds **6a** and **6b** demonstrate physicochemical profiles consistent with strong protein binding affinity; however, their high molecular weights and lipophilicity may limit oral bioavailability. Compound **7c**, despite favorable membrane permeability indices, suffers from poor solubility and excessive lipophilicity. Among the four candidates, compound **7e** appears to present the most balanced profile, with compliance with Lipinski criteria, moderate TPSA, adequate hydrogen bonding potential, and a favorable logP value, thus warranting further investigation as a potential lead compound (Figure 5).

### 2.4. Structure–Activity Relationship (SAR)

When SAR is evaluated for AR inhibitory activity, the position of the chlorine atoms and the methyl groups in the benzimidazole ring is found to be influential. The electron-withdrawing effect of the chlorine atoms on the phenyl ring can increase the polarization of the acetamide group and contribute to hydrogen bonding interactions in the AR catalytic region. Furthermore, the chlorine substituent can increase lipophilicity, thereby enhancing hydrophobic interactions. Compound **6b**, a monomethyl derivative bearing a 2,5-dichloro substituent, exhibits the highest AR inhibitory activity. The presence of a chlorine substituent in the middle position may allow the compound to exhibit a more favorable orientation to the enzyme’s binding site. On the other hand, compound **6d**, bearing a 2,6-dichloro substituent, shows low activity. This decrease in activity may be due to increased steric hindrance. Compounds containing the dimethylbenzimidazole structure exhibit a different SAR relationship. Compound **7c**, bearing the 3,4-dichlorophenyl substituent, is the most effective derivative. This suggests that the additional methyl group can enhance hydrophobic interaction. The low activity of compounds **7a** and **7b** indicates that increasing the lipophilic volume of the benzimidazole moiety does not homogeneously increase AR inhibition. Compound **7e**, bearing the 4-((1*H*-imidazole-1-yl)methyl)phenyl acetamide structure, is also moderately effective. The imidazole ring contributed to additional polar and π-type interactions with the enzyme.

When evaluating the SAR study for α-glucosidase inhibitory activity, compound **6a**, bearing monomethyl and 3,5-dichlorophenyl groups, exhibited the highest activity. Chlorinated substituents at the *meta* position can enhance the hydrophobic character of the phenyl ring without creating steric hindrance. The low efficacy of other chlorinated derivatives indicates that the position of the chlorinated substituent is important for activity. *Ortho*-chlorinated groups may cause a decrease in activity due to steric effects. Compound **7e**, with its 5,6-dimethyl group, was the most effective derivative. The difference in the imidazole ring contributed to additional hydrogen bonds or π-type interactions in the enzyme interaction. This effect is also related to the overall structure of the compound. Another derivative, **6e**, which also has an imidazole structure, shows a lower efficacy.

## 3. Materials and Methods

### 3.1. Synthesis of Benzimidazole Oxadiazole Derivative

All the chemicals employed in the synthetic procedure (Methyl 4-formylbenzoate, 4,5-dimethylbenzene-1,2-diamine or 4-methylbenzene-1,2-diamine, hydrazine hydrate) were purchased from Sigma-Aldrich Chemicals (Sigma-Aldrich Corp., St. Louis, MO, USA). The aniline derivatives, CS_2_, NaOH were purchased from Merck Chemicals (Merck KGaA, Darmstadt, Germany). Melting points of the obtained compounds were determined by MP90 digital melting point apparatus (Mettler Toledo, Columbus, OH, USA) and were uncorrected. ^1^H-NMR and ^13^C-NMR spectra of the synthesized compounds were performed by a Bruker 400 MHz and 100 MHz digital FT-NMR spectrometer (Bruker Bioscience, Billerica, MA, USA) in DMSO-*d*_6_, respectively. Splitting patterns were designated as follows: s: singlet; d: doublet; t: triplet; m: multiplet in the NMR spectra. Coupling constants (*J*) were reported as Hertz. All reactions were monitored by thin-layer chromatography (TLC) using Silica Gel 60 F254 TLC plates (Merck KGaA, Darmstadt, Germany). Docking simulations were carried out using AutoDock Vina version 1.1.2 employing the AutoDock4 (AD4) force field scoring function, and results were visualized using UCSF Chimera (version 1.17.3) and BIOVIA Discovery Studio software packages.

Synthesis of sodium metabisulfite salt of benzaldehyde (**1**) derivative:

Methyl 4-formylbenzoate (4 g, 0.02 mol) was dissolved in ethanol (30 mL). A solution of sodium metabisulfite (4.5 g, 0.024 mol) in ethanol (20 mL) was added dropwise to this solution. After completion of the addition, the reaction mixture was stirred at room temperature for 1 h. The resulting precipitate was collected by vacuum filtration, washed with cold ethanol (2 × 10 mL), and dried under reduced pressure to afford the sodium metabisulfite adduct as a solid. The crude product was used in the next step without further purification [30]. Yield: 98%.

Synthesis of methyl 4-(5,6-dimethyl-1*H*-benzo[d]imidazole-2-yl)benzoate (**2a**) or methyl 4-(5-methyl-1*H*-benzo[d]imidazole-2-yl)benzoate (**2b**):

After dissolving 4,5-dimethylbenzene-1,2-diamine or 4-methylbenzene-1,2-diamine (0.022 mol) in DMF (10 mL), sodium metabisulfite salt of benzaldehyde derivative (7.09 g, 0.026 mol) was added and refluxed for 3–4 h. At the end of the reaction, the reaction content was poured into ice water and the product was precipitated. The resulting solid was collected by filtration, washed with water to remove residual DMF and inorganic impurities, and dried under reduced pressure to afford the crude product. The crude product was recrystallized from ethanol to give the pure product [30]. Yield: 96–95%. Literature m.p. (**2a**): 208.9 °C [31]. Found m.p. (**2a**): 206.8–209.2 °C. Literature m.p. (**2b**): 252–255 °C [32]. Found m.p. (**2b**): 250.4–253.8 °C.

Synthesis of 4-(5,6-dimethyl-1*H*-benzimidazole-2-yl)benzohydrazide (**3a**) or 4-(5-methyl-1*H*-benzimidazole-2-yl)benzohydrazide derivatives (**3b**):

Compound **2a** or **2b** (0.018 mol) and excess of hydrazine hydrate (5 mL) were dissolved in ethanol (15 mL). For 12 h, the reaction mixture was refluxed. Following the completion of the reaction, the mixture was put into ice water, and the result was filtered. The resulting solid was collected by filtration, washed with cold water, and dried under reduced pressure to afford the crude product. The crude product was recrystallized from ethanol to afford the pure product [30]. Yield: 75–80%. Literature m.p. (**3a**): 316.5 °C [31]. Found m.p. (**3a**): 315.8–317.7 °C. Literature m.p. (**3b**): 258–260 °C [32]. Found m.p. (**3b**): 257.8–260.5 °C.

Synthesis of 5-[4-(5,6-dimethyl-1*H*-benzimidazol-2-yl)phenyl]-1,3,4-oxadiazol-2-thiol (**4a**) or 5-[4-(5-methyl-1*H*-benzimidazol-2-yl)phenyl]-1,3,4-oxadiazol-2-thiol derivatives (**4b**):

Compounds **3a** or **3b** (0.031 mol) and NaOH (1.48 g, 0.037 mol) were dissolved in ethanol (150 mL). After adding carbon disulfide (2.24 mL, 0.037 mol), the reaction mixture was refluxed for 8 h. After this time, the solution was cooled and acidified to pH 4–5 with hydrochloric acid solution. The resulting precipitate was collected by filtration and recrystallized from ethanol to afford the pure product [30]. Yield: 78–81%. Literature m.p. (**4b**): 295–297 °C [32]. Found m.p. (**4b**): 296.9–298.2 °C.

Synthesis of acetylated aniline derivatives (**5a**–**5e**):

Chloroacetyl chloride (0.014 mol, 1.056 mL) was dissolved in THF and placed in an ice bath. Aniline derivatives (0.012 mol) and TEA (0.0132 mol, 1.90 mL) in THF (50 mL) were added dropwise to the chloroacetyl chloride solution. After completion of dropping the reaction mixture was stirred at room temperature for 1 h. The precipitated product was collected by filtration, washed with water, and dried under reduced pressure. The crude product was used without further purification [33]. Yield: 68–75%. 2-Chloro-*N*-(3,5-dichlorophenyl)acetamide (**5a**) Literature m.p.: 140–142 °C [34]. Found m.p.: 141.2–143.4 °C. 2-Chloro-*N*-(2,5-dichlorophenyl)acetamide (**5b**) Literature m.p.: 176–177 °C [35]. Found m.p.: 175.8–176.9 °C. 2-Chloro-*N*-(3,4-dichlorophenyl)acetamide (**5c**) Literature m.p.: 104.5–106 °C [36]. Found m.p.: 103.5–105.6 °C. 2-Chloro-*N*-(2,6-dichlorophenyl)acetamide (**5d**) Literature m.p.: 170 °C [37]. Found m.p.: 167.2–168.4 °C. 2-Chloro-*N*-[4-((1H-imidazol-1-yl)-methyl)phenyl)acetamide (**5e**) Literature m.p.: 240.5–242.8 °C [38]. Found m.p.: 241.4–243.6 °C.

Synthesis of target compounds (**6a**–**6e**, **7a**–**7e**):

Compound **4a** or **4b** (0.001 mol), acetylated aniline (0.001 mol) derivative, and potassium carbonate (0.001 mol, 0.138 g) were dissolved in acetone (30 mL) and refluxed for 6h. After completion of the reaction as monitored by TLC, the solvent was removed under reduced pressure. The resulting residue was washed with water, dried, and recrystallized from ethanol to afford the final compounds (**6a**–**6e** and **7a**–**7e**) [30].

2-((5-(4-(5-methyl-1*H*-benzo[*d*]imidazol-2-yl)phenyl)-1,3,4-oxadiazol-2-yl)thio)-*N*-(3,5-dichlorophenyl) acetamide (**6a**): Yield: 67%. M.p.: 117.6–119.4 °C. ^1^H-NMR (400 MHz, DMSO-*d*_6_): *δ*: 2.44 (3H, s, CH_3_), 4.40 (2H, s, CH_2_), 7.07 (2H, d, *J* = 7.60 Hz, Aromatic CH), 7.43 (1H, s, Aromatic CH), 7.53 (1H, d, *J* = 7.00 Hz, Aromatic CH), 7.75 (1H, s, Aromatic CH), 8.02 (2H, d, *J* = 6.64 Hz, Aromatic CH), 8.12 (1H, d, *J* = 6.16 Hz, Aromatic CH), 8.39 (2H, d, *J* = 7.80 Hz, Aromatic CH), 10.52 (1H, s, NH). ^13^C-NMR (100 MHz, DMSO-*d*_6_): *δ* = 21.81, 37.14, 117.75, 123.30, 123.68, 124.10, 124.82, 127.04, 127.42, 127.70, 132.69, 133.23, 134.60, 141.59, 149.77, 160.52, 163.94, 165.31, 166.35, 177.96. HRMS (*m*/*z*): [M + H]^+^ calcd for C_24_H_17_N_5_O_2_SCl_2_: 510.0559; found: 510.0553. Anal. calcd. For C_24_H_17_N_5_O_2_SCl_2_, C, 56.48; H, 3.36; N, 13.72. Found: C, 56.63; H, 3.35; N, 13.68.

2-((5-(4-(5-methyl-1*H*-benzo[*d*]imidazol-2-yl)phenyl)-1,3,4-oxadiazol-2-yl)thio)-*N*-(2,5-dichlorophenyl) acetamide (**6b**): Yield: 65%. M.p. = 187.2–189.6 °C. ^1^H-NMR (400 MHz, DMSO-*d*_6_): *δ*: 2.45 (3H, s, CH_3_), 4.39 (2H, s, CH_2_), 7.11 (1H, s, Aromatic CH), 7.39–7.41 (2H, m, Aromatic CH), 7.52 (1H, br.s, Aromatic CH), 7.76 (2H, br.s, Aromatic CH), 8.05 (1H, d, J = 7.92 Hz, Aromatic CH), 8.14 (2H, d, *J* = 7.08 Hz, Aromatic CH), 8.30 (1H, d, J = 7.92 Hz, Aromatic CH), 10.52 (1H, s, NH). ^13^C-NMR (100 MHz, DMSO-*d*_6_): *δ* = 21.70, 37.99, 117.82, 123.72, 124.24, 126.40, 126.52, 127.36, 127.65, 128.30, 128.91, 129.38, 129.48, 130.50, 132.63, 136.83, 140.16, 147.07, 147.33, 159.83, 162.33, 164.32, 176.79. Anal. calcd. For C_24_H_17_N_5_O_2_SCl_2_, C, 56.48; H, 3.36; N, 13.72. Found: C, 56.32; H, 3.35; N, 13.69.

2-((5-(4-(5-methyl-1*H*-benzo[*d*]imidazol-2-yl)phenyl)-1,3,4-oxadiazol-2-yl)thio)-*N*-(3,4-dichlorophenyl)acetamide (**6c**): Yield: 69%. M.p.: 192.4–194.8 °C. ^1^H-NMR (400 MHz, DMSO-d_6_): *δ*: 2.43 (3H, s, CH_3_), 4.47 (2H, s, CH_2_), 7.07 (1H, d, *J* = 7.80 Hz, Aromatic CH), 7.43 (1H, s, Aromatic CH), 7.73 (2H, d, *J* = 6.72 Hz, Aromatic CH), 7.85 (2H, d, *J* = 6.56 Hz, Aromatic CH), 8.03 (1H, s, Aromatic CH), 8.12 (2H, d, *J* = 6.20 Hz, Aromatic CH), 8.39–8.41 (1H, m, Aromatic CH), 11.19 (1H, s, NH). ^13^C-NMR (100 MHz, DMSO-*d*_6_): *δ* = 21.81, 37.20, 114.84, 115.76, 120.10, 120.54, 122.41, 124.09, 124.82, 125.05, 127.39, 127.71, 131.61, 132.68, 133.12, 135.19, 139.28, 149.79, 164.06, 165.28, 165.88, 175.20. Anal. calcd. For C_24_H_17_N_5_O_2_SCl_2_, C, 56.48; H, 3.36; N, 13.72. Found: C, 56.34; H, 3.36; N, 13.70.

2-((5-(4-(5-methyl-1*H*-benzo[*d*]imidazol-2-yl)phenyl)-1,3,4-oxadiazol-2-yl)thio)-*N*-(2,6-dichlorophenyl) acetamide (**6d**): Yield: 63%. M.p.: 203.3–205.7 °C. ^1^H-NMR (400 MHz, DMSO-*d*_6_): *δ*: 2.43 (3H, s, CH_3_), 4.24 (2H, s, CH_2_), 7.06–7.07 (2H, m, Aromatic CH), 7.50 (2H, d, *J* = 7.96 Hz, Aromatic CH), 7.79 (2H, d, *J* = 7.24 Hz, Aromatic CH), 8.11 (2H, s, Aromatic CH), 8.39 (2H, d, *J* = 7.40 Hz, Aromatic CH), 10.33 (1H, s, NH). ^13^C-NMR (100 MHz, DMSO-*d_6_*): *δ* = 21.80, 38.06, 114.87, 115.80, 117.53, 118.23, 123.75, 124.93, 127.04, 127.75, 129.55, 130.90, 132.81, 133.02, 134.32, 135.53, 149.68, 149.84, 160.50, 177.95. HRMS (*m*/*z*): [M + H]^+^ calcd for C_24_H_17_N_5_O_2_SCl_2_: 510.0553; found: 510.0557. Anal. calcd. For C_24_H_17_N_5_O_2_SCl_2_, C, 56.48; H, 3.36; N, 13.72. Found: C, 56.44; H, 3.36; N, 13.71.

2-((5-(4-(5-methyl-1*H*-benzo[*d*]imidazol-2-yl)phenyl)-1,3,4-oxadiazol-2-yl)thio)-*N*-(4-((1*H*-imidazole)-1-yl) methyl)phenyl) acetamide (**6e**): Yield: 72%. M.p.: 164.5–167.2 °C. ^1^H-NMR (400 MHz, DMSO-*d*_6_): *δ*: 2.42 (3H, s, CH_3_), 4.43 (2H, s, CH_2_), 5.33 (2H, s, CH_2_), 7.04 (1H, d, *J* = 6.84 Hz, Aromatic CH), 7.38–7.41 (3H, m, Aromatic CH), 7.51 (2H, d, *J* = 6.88 Hz, Aromatic CH), 7.61 (1H, s, Aromatic CH), 7.67 (1H, d, *J* = 6.20 Hz, Aromatic CH), 7.99 (1H, d, *J* = 6.32 Hz, Aromatic CH), 8.09 (2H, d, *J* = 6.76 Hz, Aromatic CH), 8.38 (3H, s, Aromatic CH), 10.99 (1H, s, NH). ^13^C-NMR (100 MHz, DMSO-*d*_6_): *δ* = 21.81, 37.18, 51.01, 115.94, 119.89, 123.86, 123.99, 124.52, 126.76, 127.34, 127.56, 129.25, 131.44, 132.33, 133.29, 133.65, 139.34, 150.02, 150.10, 160.66, 164.01, 165.30, 165.65, 178.45. HRMS (*m*/*z*): [M + H]^+^ calcd for C_28_H_23_N_7_O_2_S: 522.1707; found: 522.1714. Anal. calcd. For C_28_H_23_N_7_O_2_S, C, 64.48; H, 4.44; N, 18.80. Found: C, 64.29; H, 4.45; N, 18.84.

2-((5-(4-(5,6-dimethyl-1*H*-benzo[*d*]imidazol-2-yl)phenyl)-1,3,4-oxadiazol-2-yl)thio)-*N*-(3,5-dichlorophenyl) acetamide (**7a**): Yield: 69%. M.p.: 175.6–177.9 °C. ^1^H-NMR (400 MHz, DMSO-*d*_6_): *δ*: 2.31 (6H, s, CH_2_), 4.44 (2H, s, CH_2_), 7.26 (1H, d, J = 8.92 Hz, Aromatic CH), 7.66 (3H, br.s., Aromatic CH), 8.12 (3H, d, *J* = 8.60 Hz, Aromatic CH), 8.33 (2H, d, *J* = 8.20 Hz, Aromatic CH), 10.13 (1H, s, NH), 12.89 (1H, s, NH). ^13^C-NMR (100 MHz, DMSO-*d*_6_): *δ* = 20.49, 38.23, 111.98, 119.33, 121.82, 124.13, 124.97, 125.73, 126.26, 127.27, 128.36, 129.44, 131.39, 132.38, 134.19, 142.96, 150.10, 161.09, 164.58, 180.56. HRMS (*m*/*z*): [M + H]^+^ calcd for C_25_H_19_N_5_O_2_SCl_2_: 524.0709; found: 524.0703. Anal. calcd. For C_25_H_19_N_5_O_2_SCl_2_, C, 57.26; H, 3.65; N, 13.35. Found: C, 57.14; H, 3.66; N, 13.38.

2-((5-(4-(5,6-dimethyl-1*H*-benzo[*d*]imidazol-2-yl)phenyl)-1,3,4-oxadiazol-2-yl)thio)-*N*-(2,5-dichlorophenyl) acetamide (**7b**): Yield: 64%. M.p.: 159.1–162.5 °C. ^1^H-NMR (400 MHz, DMSO-*d*_6_): *δ*: 2.32 (6H, s, CH_2_), 4.25 (2H, s, CH_2_), 7.26–7.27 (1H, m, Aromatic CH), 7.65 (1H, s, Aromatic CH), 7.68–7.69 (2H, m, Aromatic CH), 8.10 (3H, d, *J* = 7.68 Hz, Aromatic CH), 8.35 (2H, d, *J* = 8.04 Hz, Aromatic CH), 10.19 (1H, s, NH). ^13^C-NMR (100 MHz, DMSO-*d*_6_): *δ* = 21.51, 37.40, 115.70, 123.97, 124.68, 124.82, 126.31, 126.58, 127.34, 127.48, 127.69, 131.48, 131.63, 132.64, 132.74, 138.51, 149.50, 149.77, 155.13, 160.83, 163.86, 179.38. HRMS (*m*/*z*): [M + H]^+^ calcd for C_25_H_19_N_5_O_2_SCl_2_: 524.0709; found: 524.0700. Anal. calcd. For C_25_H_19_N_5_O_2_SCl_2_, C, 57.26; H, 3.65; N, 13.35. Found: C, 57.18; H, 3.66; N, 13.36.

2-((5-(4-(5,6-dimethyl-1*H*-benzo[*d*]imidazol-2-yl)phenyl)-1,3,4-oxadiazol-2-yl)thio)-*N*-(3,4-dichlorophenyl)acetamide (**7c**): Yield: 67%. M.p.: 191.9–193.4 °C. ^1^H-NMR (400 MHz, DMSO-*d*_6_): *δ*: 2.31 (6H, s, CH_2_), 4.43 (2H, s, CH_2_), 7.61 (2H, d, *J* = 7.60 Hz, Aromatic CH), 7.70 (1H, s, Aromatic CH), 7.76 (2H, d, *J* = 7.48 Hz, Aromatic CH), 8.21 (1H, s, Aromatic CH), 8.24 (1H, d, *J* = 6.80 Hz, Aromatic CH), 8.34 (2H, d, *J* = 6.48 Hz, Aromatic CH), 10.86 (1H, s, NH), 13.03 (1H, s, NH). ^13^C-NMR (100 MHz, DMSO-*d*_6_): *δ* = 20.51, 37.25, 118.49, 120.68, 121.41, 123.69, 125.63, 126.36, 127.24, 127.37, 127.42, 130.19, 131.42, 132.99, 135.87, 137.91, 149.50, 150.14, 160.99, 164.02, 165.36, 180.70. Anal. calcd. For C_25_H_19_N_5_O_2_SCl_2_, C, 57.26; H, 3.65; N, 13.35. Found: C, 57.44; H, 3.64; N, 13.35.

2-((5-(4-(5,6-dimethyl-1*H*-benzo[*d*]imidazol-2-yl)phenyl)-1,3,4-oxadiazol-2-yl)thio)-*N*-(2,6-dichlorophenyl) acetamide (**7d**): Yield: 63%. M.p.: 163.7–165.9 °C. ^1^H-NMR (400 MHz, DMSO-*d*_6_): *δ*: 2.33 (6H, s, CH_2_), 4.38 (2H, s, CH_2_), 7.26 (1H, d, J = 8.72 Hz, Aromatic CH), 7.68 (3H, br.s., Aromatic CH), 8.13 (3H, d, J = 8.32 Hz, Aromatic CH), 8.33 (2H, d, J = 7.96 Hz, Aromatic CH). ^13^C-NMR (100 MHz, DMSO-*d*_6_): *δ* = 20.51, 37.50, 115.70, 124.95, 126.26, 126.69, 127.33, 127.47, 127.70, 130.31, 131.46, 132.55, 133.30, 134.52, 138.61, 149.51, 149.81, 160.84, 164.30, 179.48. HRMS (*m*/*z*): [M + H]^+^ calcd for C_25_H_19_N_5_O_2_SCl_2_: 524.0709; found: 524.0706. Anal. calcd. For C_25_H_19_N_5_O_2_SCl_2_, C, 57.26; H, 3.65; N, 13.35. Found: C, 57.29; H, 3.66; N, 13.34.

2-((5-(4-(5,6-dimethyl-1*H*-benzo[*d*]imidazol-2-yl)phenyl)-1,3,4-oxadiazol-2-yl)thio)-*N*-(4-((1*H*-imidazole)-1-yl) methyl)phenyl) acetamide (**7e**): Yield: 70%. M.p.: 134.9–137.4 °C. ^1^H-NMR (400 MHz, DMSO-*d*_6_): *δ*: 2.33 (6H, s, CH_2_), 4.20 (2H, s, CH_2_), 5.29 (2H, s, CH_2_), 7.24–7.28 (2H, m, Aromatic CH), 7.41–7.44 (1H, m, Aromatic CH), 7.58–7.78 (5H, m, Aromatic CH), 8.13 (3H, d, J = 7.60 Hz, Aromatic CH), 8.33 (2H, d, J = 7.60 Hz, Aromatic CH), 12.84 (1H, s, NH). ^13^C-NMR (100 MHz, DMSO-*d*_6_): *δ* = 21.51, 37.94, 49.42, 119.33, 120.14, 122.23, 125.63, 126.38, 126.51, 127.25, 127.48, 127.73, 128.39, 128.92, 129.30, 131.41, 133.36, 134.70, 138.02, 138.93, 150.16, 160.99, 164.11, 180.71. HRMS (*m*/*z*): [M + H]^+^ calcd for C_29_H_25_N_7_O_2_S: 536.1863; found: 536.1867. Anal. calcd. For C_29_H_25_N_7_O_2_S, C, 65.03; H, 4.70; N, 18.30. Found: C, 64.82; H, 4.71; N, 18.26.

### 3.2. Biological Activity

#### 3.2.1. Aldose Reductase Assay

As reported in the previous literature, the AR enzyme was purified from sheep liver. The experimental studies were performed using certain modifications of the methods described in the relevant sources [39]. By tracking the drop in absorbance at the 340 nm wavelength caused by NADPH oxidation during the reaction, the AR enzyme’s activity was assessed. This was accomplished by preparing 0.8 M sodium phosphate buffer (pH 5.5), 0.11 mM NADPH, 4.7 mM DL-glyceraldehyde (substrate), and a suitable volume of enzyme solution. The inhibitory effect of the tested compounds on AR was quantitatively assessed using the spectrophotometric data collected under these conditions.

#### 3.2.2. α-GLY Assay

The α-GLY enzyme activity was evaluated using p-nitrophenyl-α-D-glucopyranoside (pNPG) as a substrate according to the method reported by Tao and colleagues [40]. In the experimental application, 100 µL of phosphate buffer was first mixed with 20 µL of enzyme solution (pH 6.8, 0.15 U/mL) and the prepared sample solution in the concentration range of 0.01–1 mg/mL (10–100 µL). To ensure the effectiveness of enzyme inhibition, different sample solutions were prepared and tested in phosphate buffer. The mixture was pre-incubated at 35 °C for 12 min before starting the reaction. Then 50 µL of pNPG solution (5.0 mM, pH 7.4) was added to start the reaction and the mixture was incubated at 37 °C. The amount of p-nitrophenol formed by the enzymatic hydrolysis at the end of the reaction was determined by the absorbance change measured at 405 nm.

#### 3.2.3. *In Vitro* Inhibition Studies

The newly synthesised benzimidazole-oxadiazole derivatives (**6a**–**6e**, **7a**–**7e**) were tested at different concentrations against AR and α-GLY enzymes and their inhibitory activity was evaluated. The inhibitory activity analyses were performed at different concentrations for each compound. The IC_50_ values of the derivatives were calculated in the Microsoft Excel environment using activity-percent concentration plots showing the effect of the inhibitor concentration against the activity of the enzyme of interest. These calculations were performed in the concentration ranges of 1–50 nM for AR and 1–400 µM for α-GLY. In addition, the inhibition profiles of the compounds were analysed based on enzyme kinetics using the Lineweaver–Burk double inversion method and, accordingly, the inhibition constants (K_I_) were determined along with the types of inhibition [41,42]. These analyses provide detailed information on the interaction mechanisms and selectivity levels of the compounds with the target enzymes.

### 3.3. Molecular Docking Study

The three-dimensional structures of the target proteins employed in the molecular docking investigations were retrieved from the Protein Data Bank (RCSB PDB). Docking simulations were carried out using AutoDock Vina version 1.1.2 employing the AutoDock4 (AD4) force field scoring function, and results were visualized using UCSF Chimera (version 1.17.3) and BIOVIA Discovery Studio software packages [43,44]. The superimposition of ligand conformations was performed using UCSF Chimera (version 1.17.3) to evaluate binding pose consistency and structural alignment. Ligand molecules were initially constructed using ChemDraw 12.0, followed by geometric optimization. Energy minimization of the ligands was performed at the MM2 force field level employing Chem3D Pro to obtain the most energetically favorable conformations.

The crystal structures of the receptor proteins (PDB IDs: 3WY1 and 4JIR) were downloaded from the RCSB database and prepared for docking calculations using the UCSF Chimera input module. During the protein and ligand preparation process, steric clashes were eliminated, hydrogen atoms and partial charges were assigned, missing residues and side chains were corrected, and incomplete loop regions were reconstructed. Additionally, crystallographic water molecules located more than 5 Å away from the binding region were removed. The docking parameters used were as for 4JIR: Grid Center (X, Y, Z) = (−0.793, 0.213, 16.754); Grid Box Size (X, Y, Z) = (49.358 × 39.85 × 49.674 Å); Exhaustiveness = 8; Number of binding modes = 10; Maximum energy difference = 3 kcal/mol. 3WY1: Grid Center (X, Y, Z) = (−10.366, −12.4775, 9.4655); Grid Box Size (X, Y, Z) = (47.128 × 51.931 × 76.895 Å); Exhaustiveness = 8; Number of binding modes = 10; Maximum energy difference = 3 kcal/mol. The active binding pockets of the proteins were identified, optimized, and used as docking sites. Visualization and analysis of the resulting two-dimensional and three-dimensional ligand–protein interaction poses were conducted using BIOVIA Discovery Studio 2021 Client (BIOVIA, San Diego, CA, USA) [45]. The accuracy and consistency of the docking results were assessed by calculating root mean square deviation (RMSD) values using the MatchMaker tool in UCSF Chimera. Docked conformations were superimposed onto the corresponding reference crystal structures, and RMSD values below 2.0 Å were accepted as indicative of reliable and successful docking, confirming agreement with experimentally resolved binding modes.

### 3.4. ADME Analysis

In silico ADME profiling of the synthesized compounds (**6a**–**6e** and **7a**–**7e**) was carried out using the SwissADME web-based platform. The physicochemical properties, pharmacokinetic parameters, drug-likeness, and medicinal chemistry characteristics of the selected compounds were evaluated using the SwissADME web server (http://www.swissadme.ch (accessed on 10 May 2026)). Canonical SMILES notations for all compounds were generated with ChemDraw and subsequently uploaded to the SwissADME server for analysis. The computational predictions included key physicochemical and pharmacokinetic parameters such as lipophilicity, drug-likeness, absorption and distribution characteristics, topological polar surface area (TPSA), the number of rotatable bonds, and compliance with Lipinski’s rule of five [28,46].

## 4. Conclusions

In this study, new benzimidazole-oxadiazole derivative compounds (**6a**–**6e**, **7a**–**7e**) were synthesized. α-GLY and AR inhibition of the synthesized compounds were investigated. α-GLY and AR inhibition of the synthesized compounds were investigated. When the results of *in vitro* enzyme inhibition tests (AR and α-GLY) were examined, compounds **6a**, **6b,** and **6c** showed higher activity in AR inhibition than the reference drug epalrestat (IC_50_: 0.78 ± 0.0 μM; *K*_I_: 0.74 ± 0.0 μM) with *K*_I_ values of 8.6 ± 0.4, 3.5 ± 0.3 and 6.3 ± 0.5 nM, respectively. The compounds **6a** and **7e** that emerged first in α-GLY inhibition had *K*_I_ values of 5.8 ± 0.4 and 7.9 ± 0.8 µM, respectively. They showed very high inhibition compared to the reference drug Acarbose (IC_50_: 128.4 µM; *K*_I_: 96.2 ± 5.7 µM). When the results of the two assays were evaluated together, compounds **6a** and **7e** emerged as the most promising derivatives, appearing to be strong drug candidates that could be the subject of future anti-diabetes studies. This study has demonstrated that the newly synthesized molecules possess highly potent inhibitory properties, particularly against the AR enzyme. Compounds **6a**, **6b**, **7c**, and **7e** are compounds that stand out both experimentally and theoretically and may be considered candidates for further pharmacological studies. Additionally, some compounds have shown promising activity in terms of α-GLY inhibition. In future studies, it is recommended to thoroughly investigate the pharmacokinetic parameters of these molecules, including their toxicity profiles, intracellular bioavailability, and metabolic stability. Furthermore, further strengthening the docking results with molecular dynamics simulations would provide an important contribution. A limitation of the present study is that the aldose reductase enzyme was purified from sheep liver rather than human tissue. However, sheep aldose reductase has been extensively used as an experimental model for evaluating aldose reductase inhibitors. Moreover, epalrestat, a clinically approved aldose reductase inhibitor used for the treatment of diabetic peripheral neuropathy, has been reported as a potent inhibitor of AKR1B1 and AKR1B10 [47]. The successful inhibition of the sheep liver enzyme by epalrestat supports the suitability of this enzyme source for preliminary inhibitor screening studies. The use of human liver-derived aldose reductase is considerably restricted by ethical and practical limitations associated with human tissue procurement. Nevertheless, further studies using recombinant human AKR1B1 are warranted to confirm the translational relevance of the identified compounds.

## Figures and Tables

**Table 1:** The inhibitory potential of new benzimidazole oxadiazole derivatives against AR and α-GLY as IC_50_ and *K*_I_ values.

Compound ID	AR ^[a]^		α-GLY ^[b]^	
	*IC*_50_ (nM)	*K_I_* (nM)	*IC*_50_ (μM)	*K_I_* (μM)
**6a**	10.01 ± 0.8	8.6 ± 0.4	5.9 ± 0.4	5.8 ± 0.4
**6b**	4.93 ± 0.3	3.5 ± 0.3	>400	>400
**6c**	10.99 ± 0.6	6.3 ± 0.5	>400	>400
**6d**	12.13 ± 0.8	9.3 ± 0.7	279.3 ± 18.3	276.2 ± 25.2
**6e**	14.53 ± 0.7	13.7 ± 0.9	>400	>400
**7a**	14.19 ± 0.6	11.9 ± 1.1	163.2 ± 12.5	133.7 ± 27.8
**7b**	19.22 ± 0.8	27.7 ± 2.0	43.5 ± 1.4	35.2 ± 3.3
**7c**	5.42 ± 0.3	7.4 ± 0.6	32.6 ± 1.2	26.4 ± 2.13
**7d**	10.20 ± 0.6	19.9 ± 1.1	43.5 ± 1.8	35.2 ± 27.9
**7e**	9.01 ± 0.2	13.1 ± 1.1	9.8 ± 0.7	7.9 ± 0.8
**Epalrestat**	0.78 ± 0.0	0.74 ± 0.0	-	-
**Acarbose**	-	-	128.4 ± 7.5	96.2 ± 5.7

^[a]^ Aldose reductase, ^[b]^ α-Glycosidase.

**Table 2:** Docking scores (kcal/mol) for the compounds **6a**–**6e**, **7a**–**7e**.

	Docking Score (kcal/mol)	
Compounds	4JIR (Aldose Reductase)	3WY1 (α-Glycosidase)
**6a**	−11.5	−8.2
**6b**	−11.4	−7.3
**6c**	−11.5	−8.4
**6d**	−11.2	−7.7
**6e**	−11.3	−9.4
**7a**	−11.5	−8.2
**7b**	−11.4	−9.6
**7c**	−11.7	−9.2
**7d**	−11.3	−8.6
**7e**	−11.5	−9.5
**Epalrestat**	−7.3	-
**Acarbose**		−5.0

**Table 3:** Hydrophobic Interactions, Hydrogen Bonds, π-Cation Interactions, π-Stacking, and Halogen.

Hydrophobic Interactions							
	Residue	AA	Distance		Residue	AA	Distance
3WY1-**6a**	678B	ILE	3.58	3WY1-**7e**	678B	ILE	3.66
	829B	PHE	3.49		829B	PHE	3.72
	829B	PHE	3.09		829B	PHE	3.14
	865B	ASP	3.93		865B	ASP	3.84
	867B	VAL	3.64		867B	VAL	3.35
	921B	TYR	3.11		872B	ARG	3.78
	929B	PHE	3.87		921B	TYR	2.98
4JIR-**6b**	21A	TRP	3.37	4JIR-**7e**	21A	TRP	3.20
	210A	TYR	3.58		112A	TRP	3.62
	210A	TYR	3.62		210A	TYR	3.86
	229A	LEU	3.77		210A	TYR	3.91
	263A	LYS	3.37		213A	LEU	3.48
					246A	ALA	3.62
					263A	LYS	3.43
					269A	ARG	3.59
**Hydrogen Bonds**							
	Residue	AA	Distance H-A	Distance D-A	Donor Angle		
3WY1-**6a**	865B	ASP	2.68	3.16	110.64		
3WY1-**7e**	865B	ASP	2.40	2.92	112.34		
	932B	ARG	3.41	3.73	101.39		
4JIR-**6b**	263A	LYS	2.54	3.46	155.35		
	269A	ARG	2.41	3.13	129.61		
4JIR-**7e**	263A	LYS	2.16	3.13	166.84		
	269A	ARG	2.53	2.97	107.02		
**π-Cation Interactions**							
	Residue	AA	Distance	Ligand Group			
3WY1-**6a**	932B	ARG	4.42	Aromatic			
3WY1-**7e**	932B	ARG	4.24	Aromatic			
4JIR-**6b**	269A	ARG	3.65	Aromatic			
4JIR-**7e**	269A	ARG	4.05	Aromatic			
**π-Stacking**							
	Residue	AA	Distance	Angle	Stacking Type		
4JIR-**6b**	210A	TYR	3.67	2.31	P		
4JIR-**7e**	210A	TYR	3.56	2.33	P		
**Halogen Bonds**							
	Residue	AA	Distance	Donor Angle	Acceptor Angle		
4JIR-**6b**	273A	ASN	3.39	156.42	95.27		
	273A	ASN	3.31	141.49	101.12		

**Table 4:** Physicochemical and ADME properties of synthesized compounds **6a**, **6b**, **7c**, and **7e**.

Physicochemical Property					
	6a	6b	7c	7e	Comment
Molecular Weight (MW)	509.05	509.05	522.07	535.18	Contain hydrogen atoms. Optimal: 100~600
Volume	468.09	468.09	491.69	532.32	Van der Waals volume
Density	1.087	1.087	1.062	1.005	Density = MW/Volume
nHA	7.0	7.0	6.0	9.0	Number of hydrogen bond acceptors. Optimal: 0~12
nHD	2.0	2.0	1.0	2.0	Number of hydrogen bond donors. Optimal: 0~7
nRot	7.0	7.0	7.0	9.0	Number of rotatable bonds. Optimal: 0~11
nRing	5.0	5.0	5.0	6.0	Number of rings. Optimal: 0~6
MaxRing	9.0	9.0	9.0	9.0	Number of atoms in the biggest ring. Optimal: 0~18
nHet	10.0	10.0	9.0	10.0	Number of heteroatoms. Optimal: 1~15
fChar	0.0	0.0	0.0	0.0	Formal charge. Optimal: −4~4
nRig	28.0	28.0	28.0	33.0	Number of rigid bonds. Optimal: 0~30
Flexibility	0.25	0.25	0.25	0.273	Flexibility = nRot/nRig
Stereo Centers	0.0	0.0	0.0	0.0	Stereo Centers. Optimal: ≤2
TPSA	96.7	96.7	84.67	114.52	Topological Polar Surface Area. Optimal: 0~140
logS	−6.69	−6.69	−7.08	−5.26	The logarithm of aqueous solubility value.
logP	5.459	5.459	6.123	3.635	The logarithm of the n-octanol/water distribution coefficients at pH = 7.4.
logD7.4	4.315	4.315	4.225	3.183	The logarithm of the n-octanol/water distribution coefficients
pka (Acid)	10.836	10.836	10.38	9.957	Acid-base dissociation constant (pKa) value represents the strength of a drug molecule’s acidity or basicity.
pka (Base)	2.724	2.724	3.044	4.742	Acid-base dissociation constant (pKa) value represents the strength of a drug molecule’s acidity or basicity.
Medicinal Chemistry					
	**6a**	**6b**	**7c**	**7e**	
Lipinski Rule	Rejected	Rejected	Rejected	Accepted	
Pfizer Rule	Accepted	Accepted	Accepted	Accepted	
GSK Rule	Rejected	Rejected	Rejected	Rejected	
GoldenTriangle	Rejected	Rejected	Rejected	Rejected	

## Data Availability

The data supporting the findings of this study are provided in the manuscript and the Supplementary Information Files.

## Supplementary Materials

The following supporting information can be downloaded at: https://www.mdpi.com/article/10.3390/molecules31152635/s1, ^1^H NMR, ^13^C NMR spectra of compounds **6a**–**6e**, **7a**–**7e** are included in the supporting information file.

## Abbreviations

The following abbreviations are used in this manuscript:

α-GLY	α-glucosidase
AR	Aldose reductase
ADME	Absorption, distribution, metabolism, elimination
DM	Diabetes mellitus
WHO	World Health Organization
ARIs	Aldose reductase inhibitors
ROS	Reactive oxygen species
AGE	Advanced glycation end products
ARI	Aldose reductase inhibitor
GIs	Glucosidase inhibitors
KI	Inhibition constants
TPSA	Topological polar surface area
SAR	Structure–Activity Relationship
TLC	Thin-layer chromatography
pNPG	p-nitrophenyl-α-D-glucopyranoside
PDB	Protein Data Bank
RMSD	Root mean square deviation
