# Supplementary material for: Design, Synthesis, In Silico ADME, Toxicity Prediction and Molecular Docking Studies of Benzimidazole-Oxadiazole Derivatives for α-Glucosidase and Aldose Reductase Pathways as Potent Anti-Diabetic Agents
Source: Molecules. 2026 Jul 29;31(15):2635. doi: 10.3390/molecules31152635 (PMC13468218; doi:10.3390/molecules31152635)
Supplement: Supplementary file 1 [file molecules-31-02635-s001.zip › molecules-4376777-supplementary.pdf]

Design, Synthesis, *In Silico* ADME, Toxicity Prediction and Molecular Docking Studies of Benzimidazole-Oxadiazole Derivatives for α-Glucosidase and Aldose Reductase Pathways as Potent Anti-Diabetic Agents

Mesut Işık [1], Abdüllatif Karakaya [2,3], Ulviye Acar Çevik [4,*], Adem Necip [5], Hatice Esra Duran [6], Bilge Çiftçi [7], Yusuf Özkay [4], Zafer Asım Kaplancıklı [4,8] and Şükrü Beydemir [9]

1 Department of Bioengineering, Faculty of Engineering, Bilecik Şeyh Edebali University, 11230 Bilecik, Turkey; mesut.isik@bilecik.edu.tr
2 Department of Pharmaceutical Chemistry, Faculty of Pharmacy, Zonguldak Bulent Ecevit University, 67600 Zonguldak, Turkey; a.karakaya@beun.edu.tr
3 Department of Pharmaceutical Chemistry, Graduate School, Anadolu University, 26470 Eskisehir, Turkey
4 Department of Pharmaceutical Chemistry, Faculty of Pharmacy, Anadolu University, 26470 Eskişehir, Turkey; yozkay@anadolu.edu.tr (Y.Ö.); zakaplan@anadolu.edu.tr (Z.A.K.)
5 Department of Pharmacy Services, Vocational School of Health Services, Harran University, 63300 Şanlıurfa, Turkey; ademnecip@harran.edu.tr
6 Department of Medical Biochemistry, Faculty of Medicine, Kafkas University, 36100 Kars, Turkey; hatice.duran@kafkas.edu.tr
7 Vocational School of Health Services, Bilecik Şeyh Edebali University, 11230 Bilecik, Turkey; bilge.ciftci@bilecik.edu.tr
8 The Rectorate of Bilecik Şeyh Edebali University, 11230 Bilecik, Turkey
9 Department of Biochemistry, Faculty of Pharmacy, Anadolu Universty, 26470 Eskişehir, Turkey; sukrubeydemir@anadolu.edu.tr
* Correspondence: uacar@anadolu.edu.tr

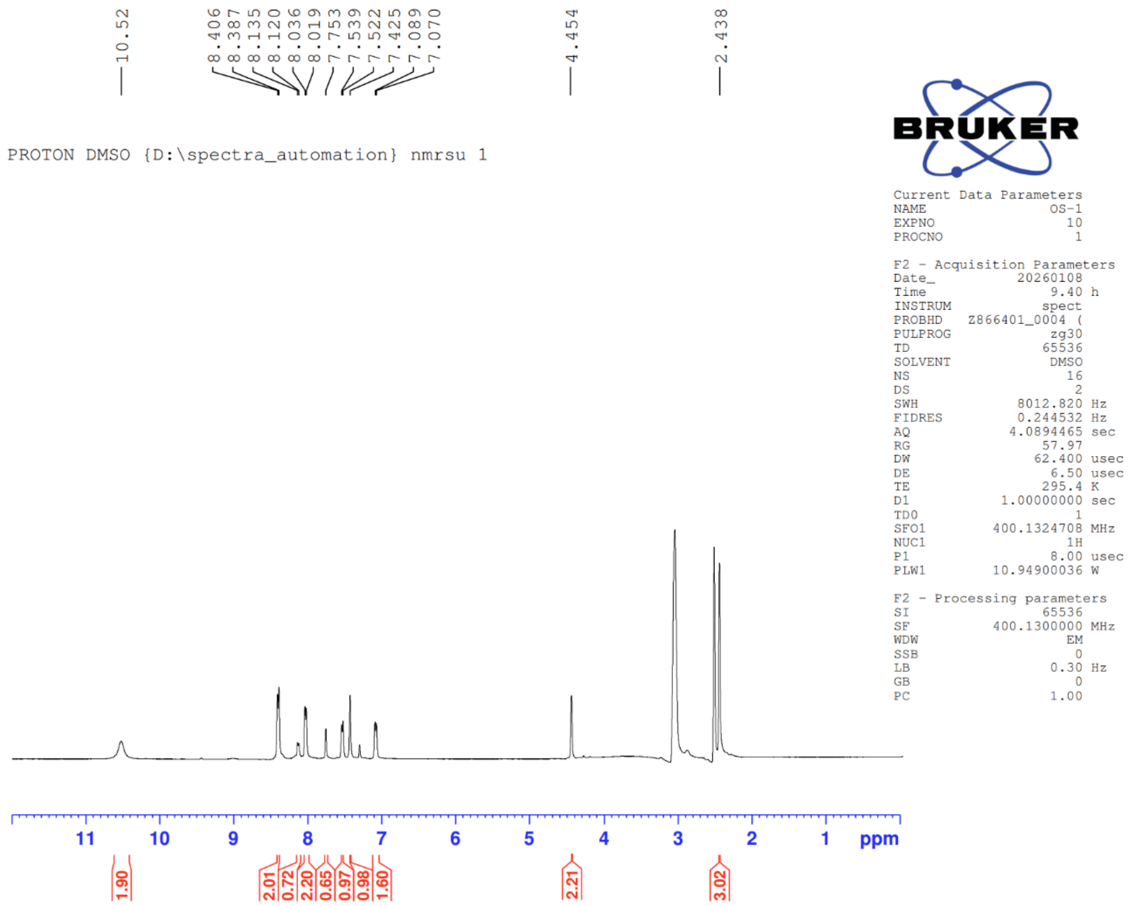

Figure S1. ^{1}H-NMR spectrum of compound **6a**

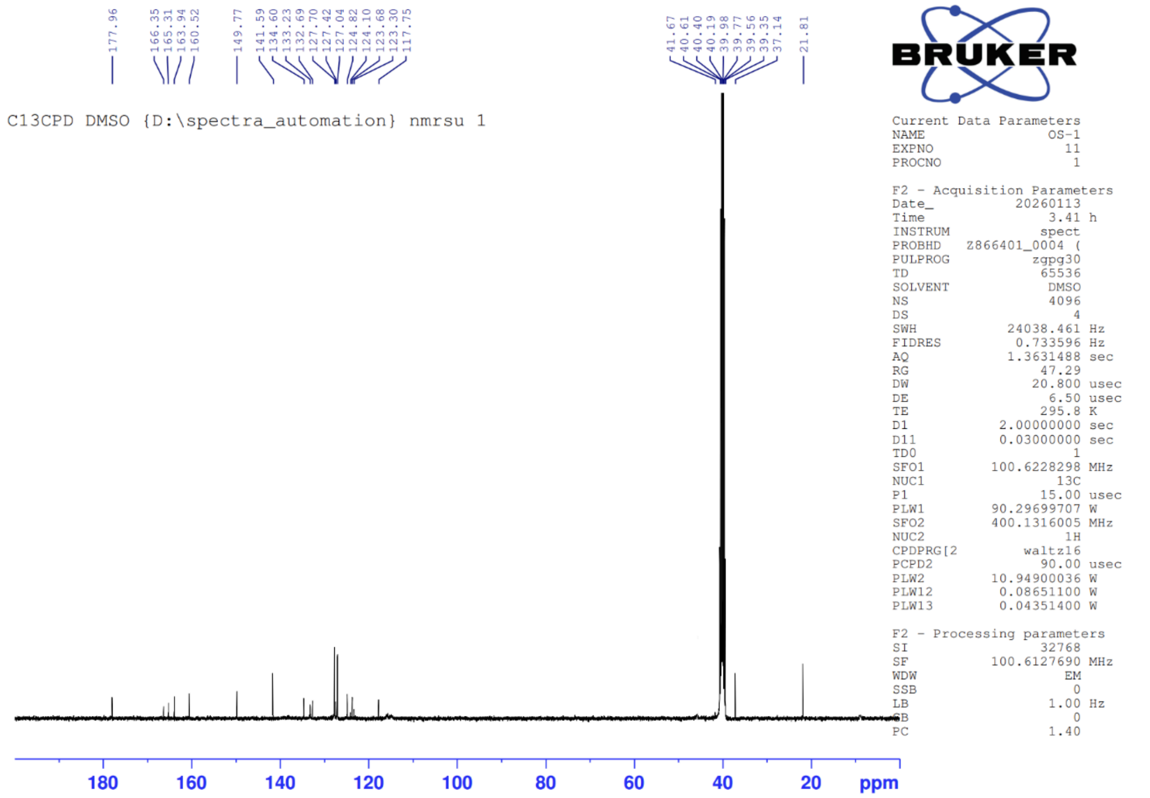

Figure S2. ^{13}C-NMR spectrum of compound **6a**

Formula Predictor Report - OS-1_175.lcd

Data File: C:\LabSolutions\Data\Analiz\uac\OS-1_175.lcd

Elmt	Val.	Min	Max	Elmt	Val.	Min	Max	Elmt	Val.	Min	Max	Elmt	Val.	Min	Max	Use Adduct
H	1	0	25	O	2	0	4	Cl	1	2	2	I	3	0	0	H
B	3	0	0	F	1	0	0	Br	1	0	0					
C	4	0	35	P	3	0	0	Ru	2	0	0					
N	3	0	7	S	2	1	1	Pd	2	0	0					

Error Margin (ppm):	5	DBE Range:	5.0 - 25.0	Electron Ions:	both
HC Ratio:	unlimited	Apply N Rule:	yes	Use MSn Info:	yes
Max Isotopes:	3	Isotope RI (%):	1.00	Isotope Res:	9000
MSn Iso RI (%):	10.00	MSn Logic Mode:	AND	Max Results:	50

Event#: 1 MS(E+) Ret. Time : 3.000 Scan# : 451

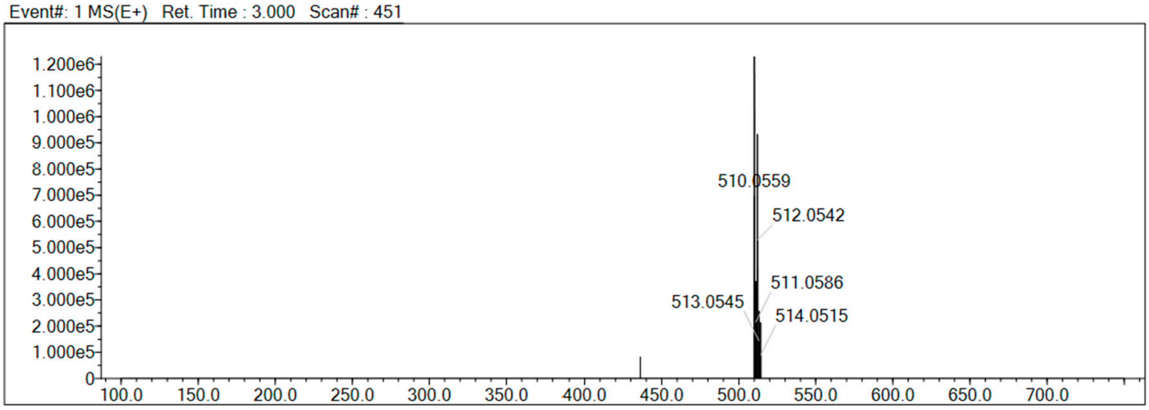

Measured region for 510.0559 m/z

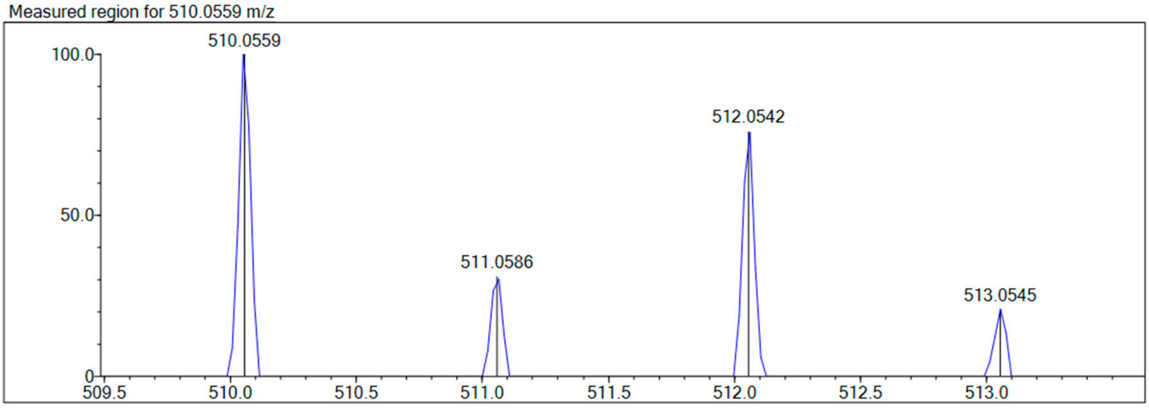

C24 H17 N5 O2 S Cl2 [M+H]+ : Predicted region for 510.0553 m/z

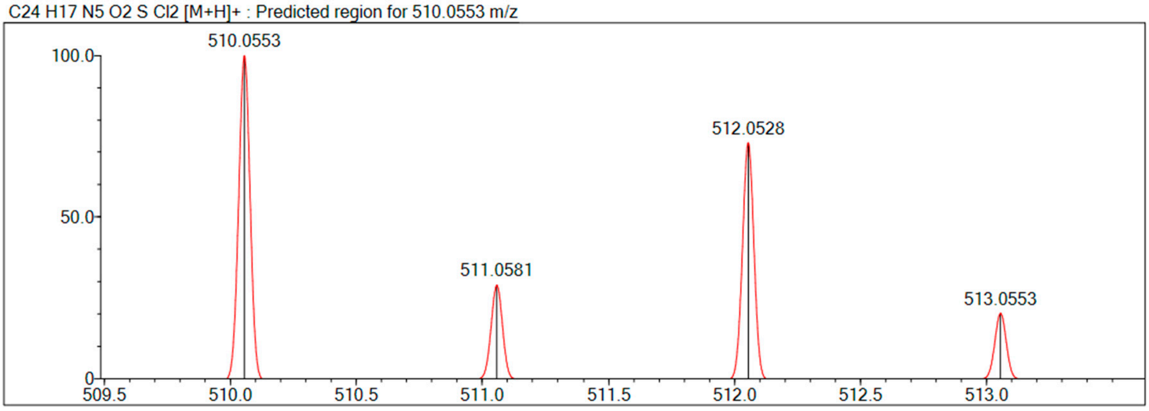

Rank	Score	Formula (M)	Ion	Meas. m/z	Pred. m/z	Df. (mDa)	Df. (ppm)	Iso	DBE
1	94.75	C24 H17 N5 O2 S Cl2	[M+H]+	510.0559	510.0553	0.6	1.18	95.18	18.0

Figure S3. Mass spectrum of compound **6a**

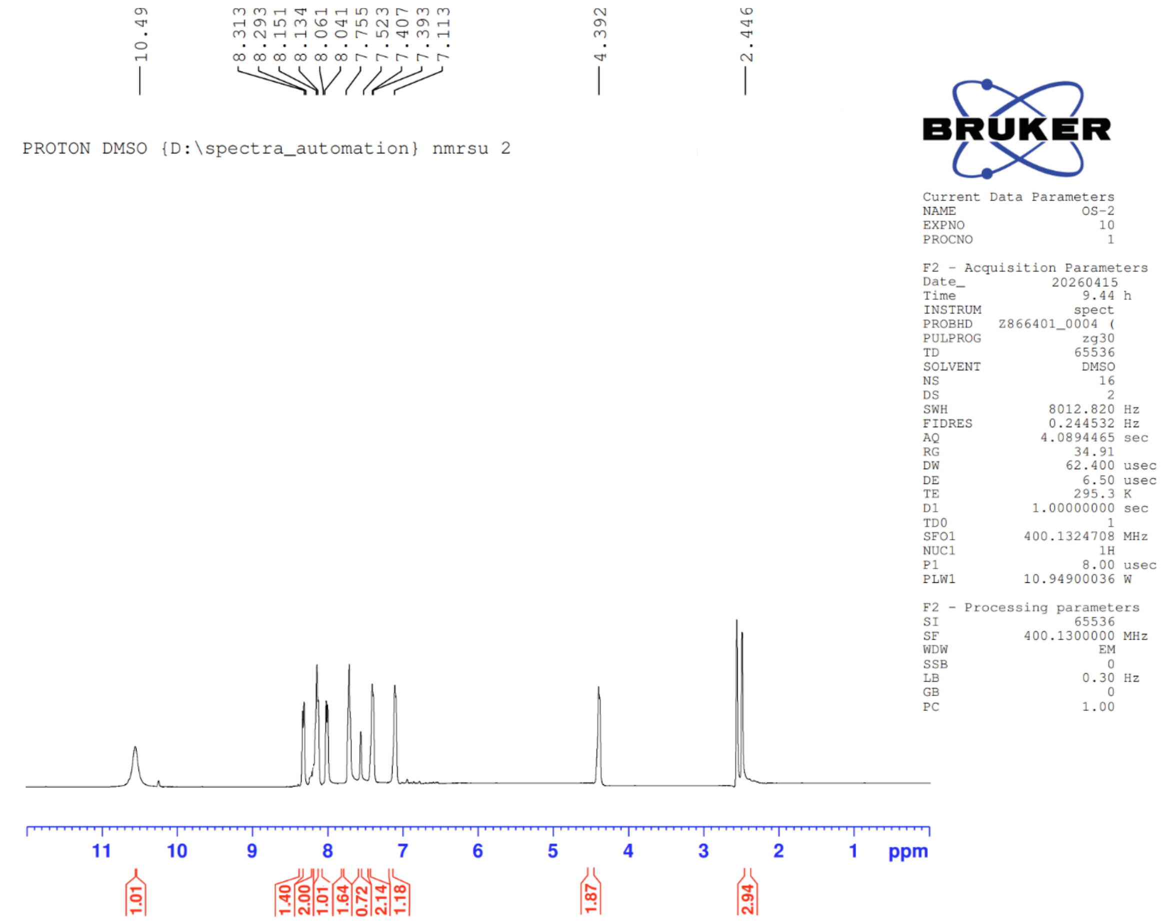

Figure S4. ^{1}H-NMR spectrum of compound **6b**

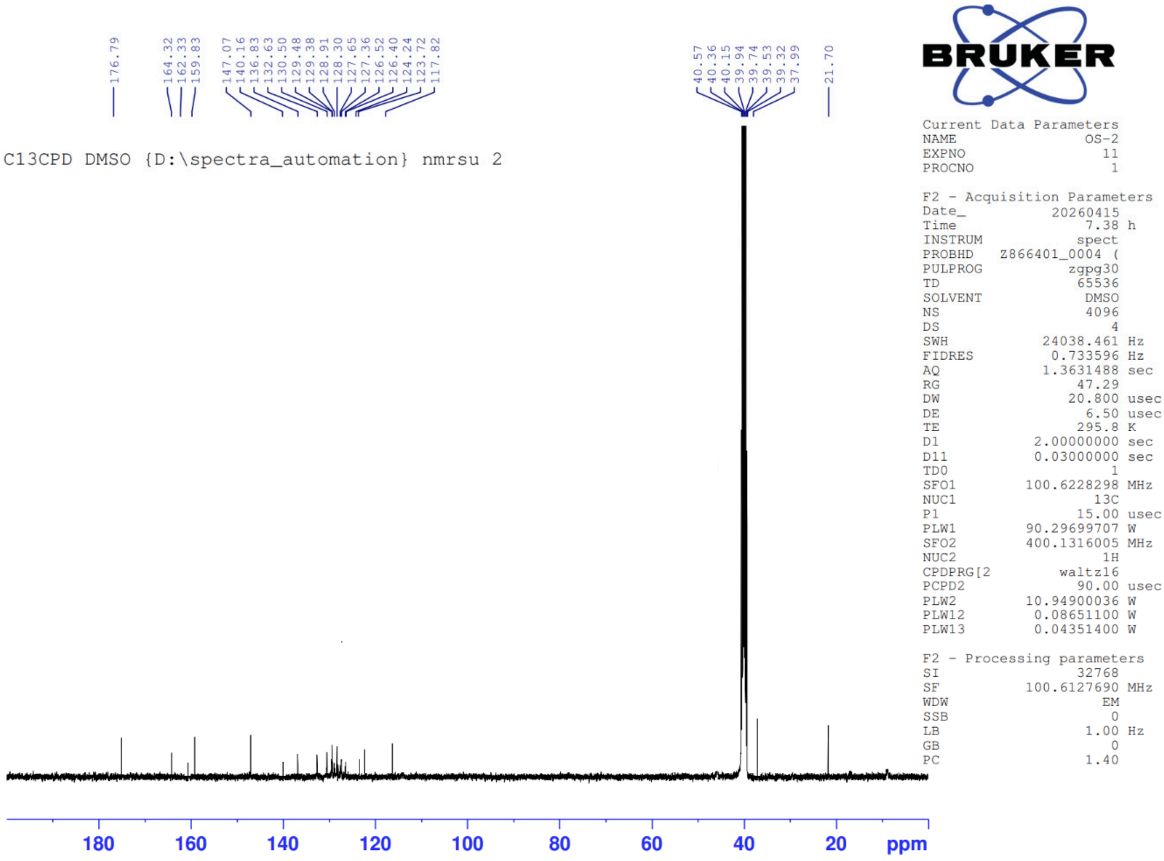

Figure S5. ^{13}C-NMR spectrum of compound **6b**

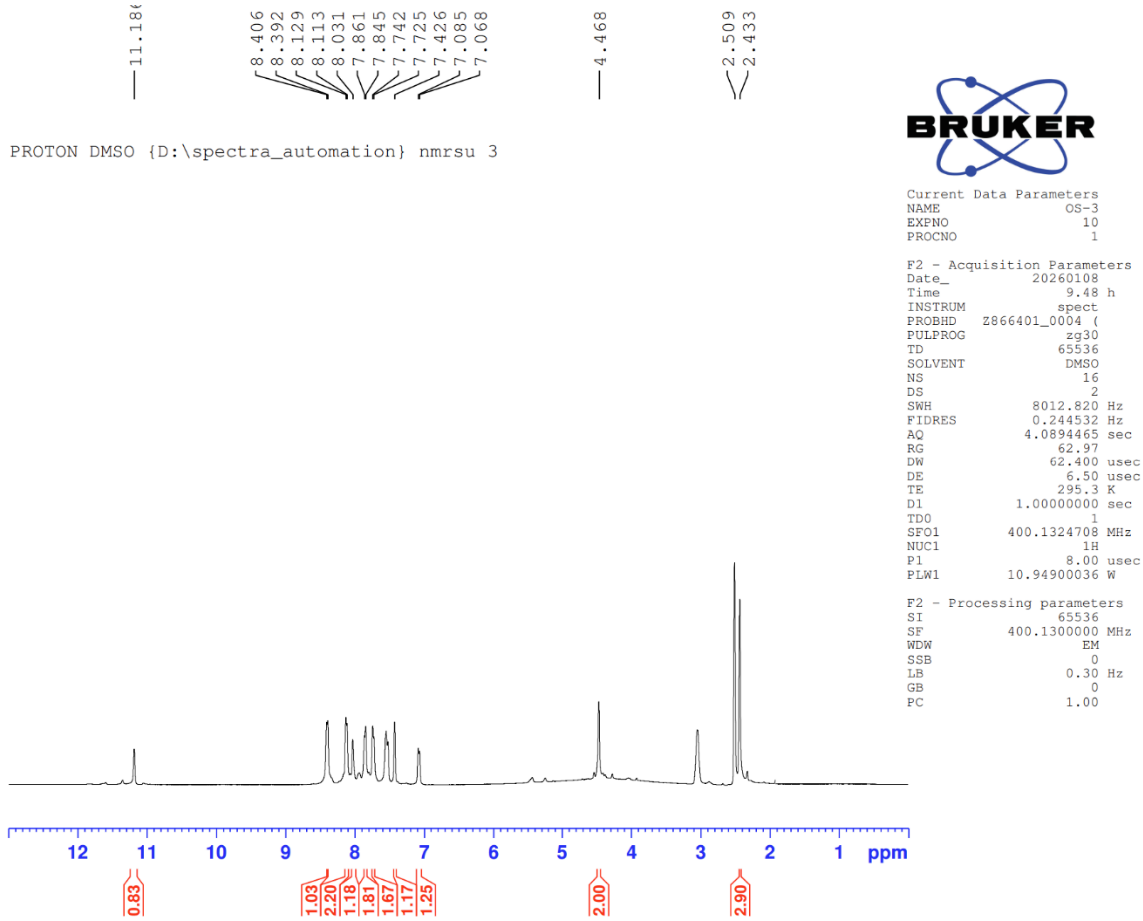

Figure S6. ^{1}H-NMR spectrum of compound **6c**

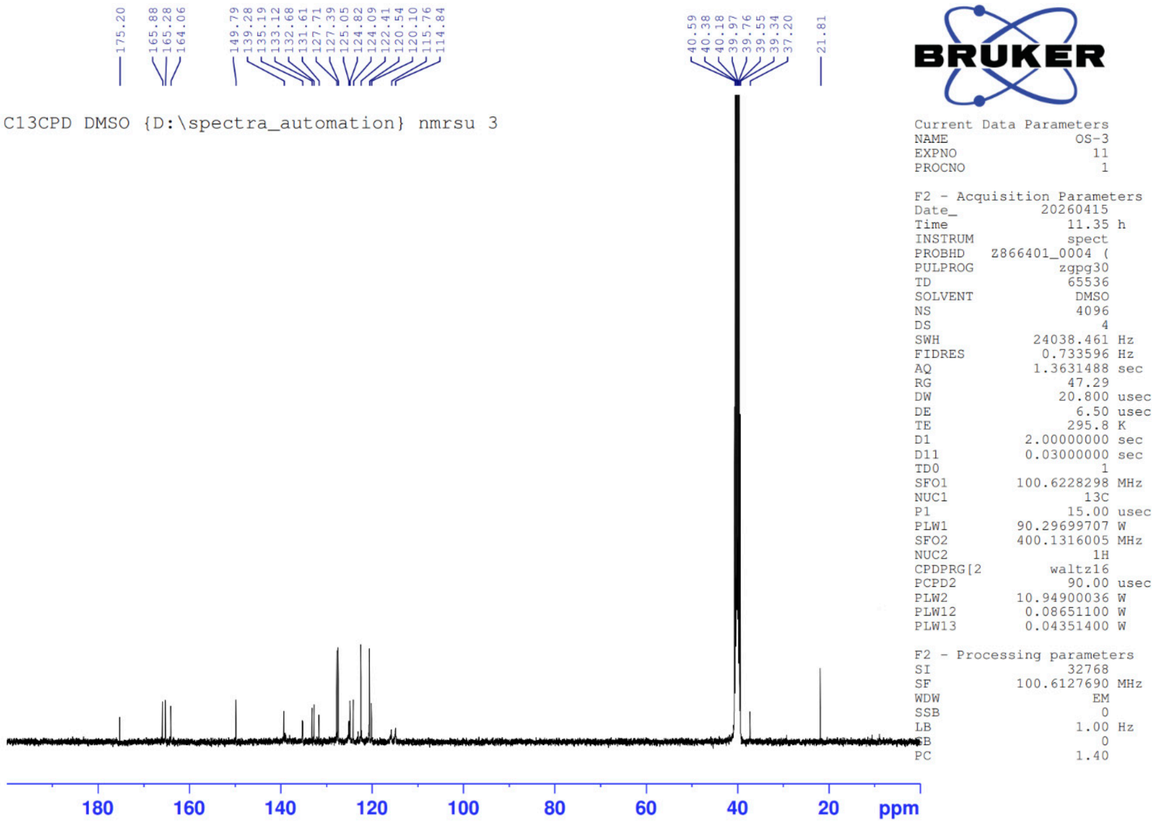

Figure S7. ^{13}C-NMR spectrum of compound **6c**

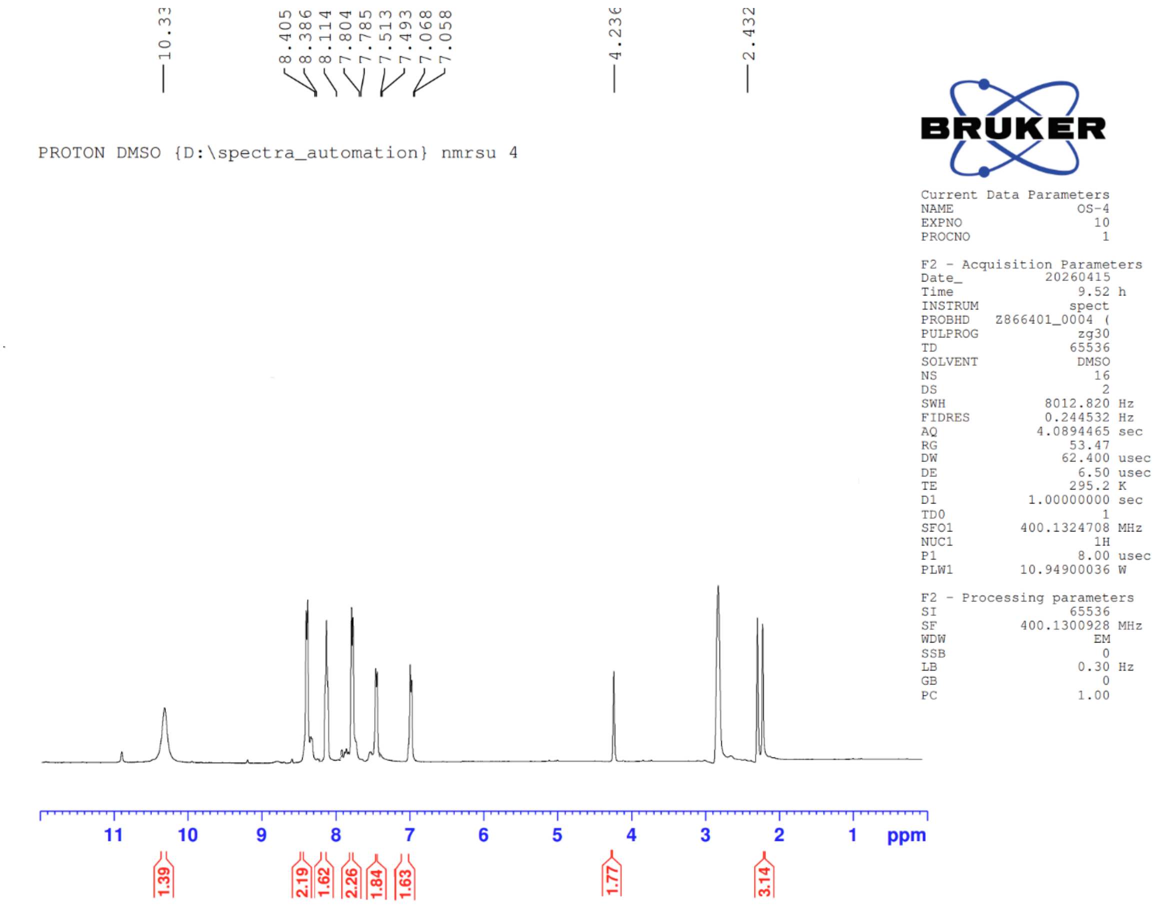

Figure S8. ^{1}H-NMR spectrum of compound **6d**

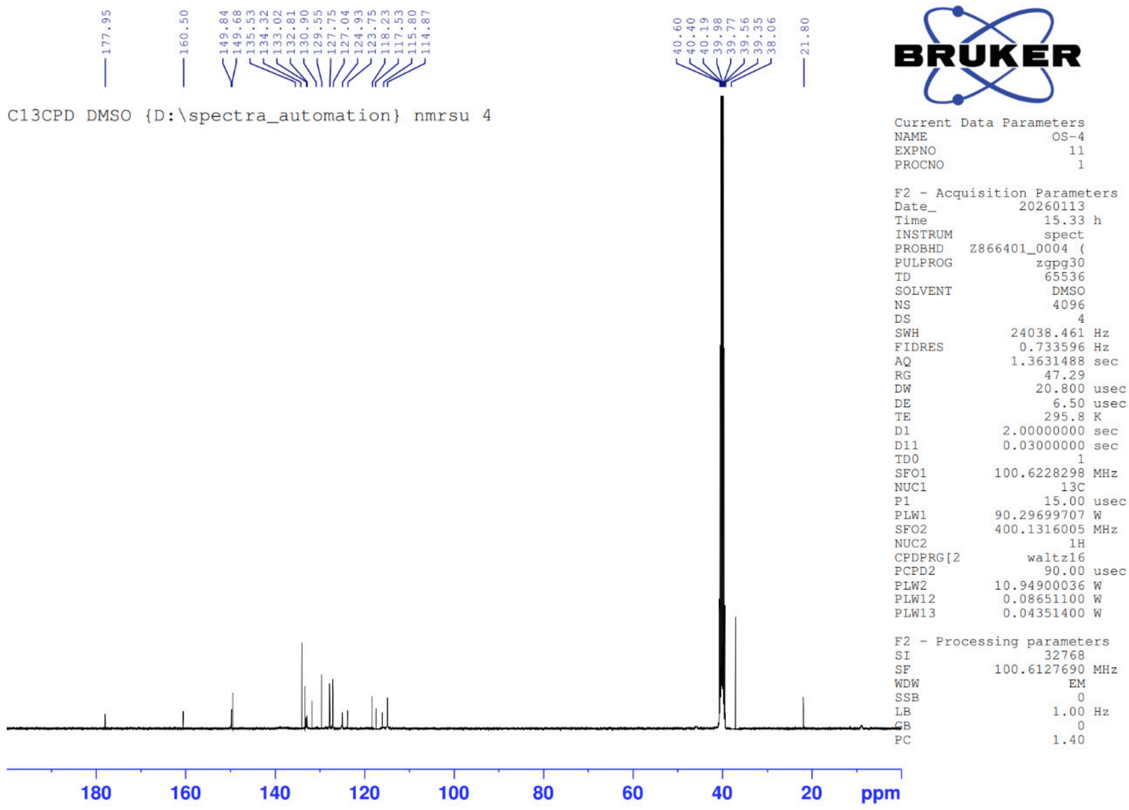

Figure S9. ^{13}C-NMR spectrum of compound **6d**

Data File: C:\LabSolutions\Data\Analiz\uac\OS-4_178.lcd

Elmt	Val.	Min	Max	Elmt	Val.	Min	Max	Elmt	Val.	Min	Max	Elmt	Val.	Min	Max	Use Adduct
H	1	0	25	O	2	0	4	Cl	1	2	2	I	3	0	0	H
B	3	0	0	F	1	0	0	Br	1	0	0					
C	4	0	35	P	3	0	0	Ru	2	0	0					
N	3	0	7	S	2	1	1	Pd	2	0	0					

Error Margin (ppm): 5
HC Ratio: unlimited
Max Isotopes: 3
MSn Iso RI (%): 10.00

DBE Range: 5.0 - 25.0
Apply N Rule: yes
Isotope RI (%): 1.00
MSn Logic Mode: AND

Electron Ions: both
Use MSn Info: yes
Isotope Res: 9000
Max Results: 50

Event#: 1 MS(E+) Ret. Time : 2.147 Scan# : 323

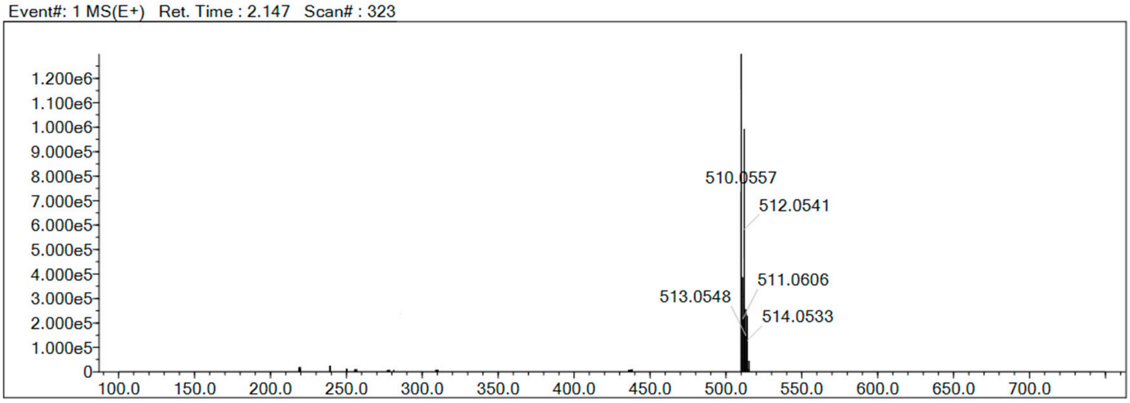

Measured region for 510.0557 m/z

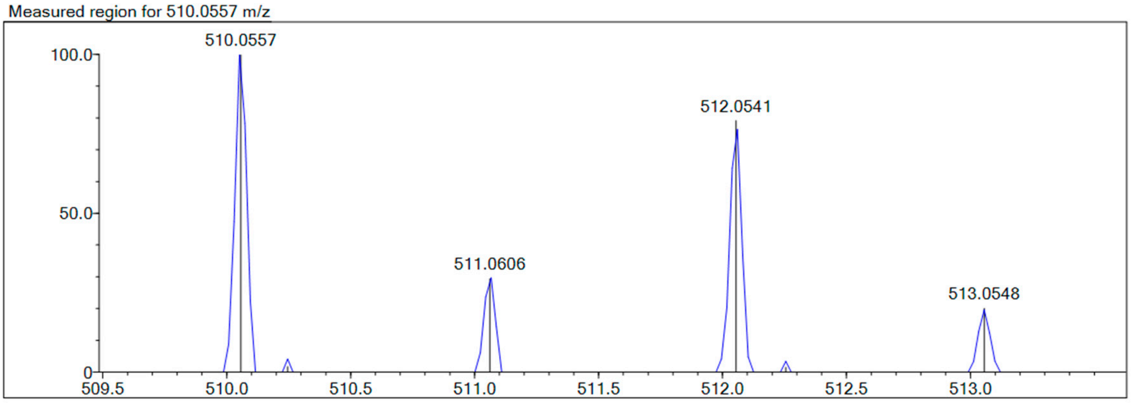

C24 H17 N5 O2 S Cl2 [M+H]+ : Predicted region for 510.0553 m/z

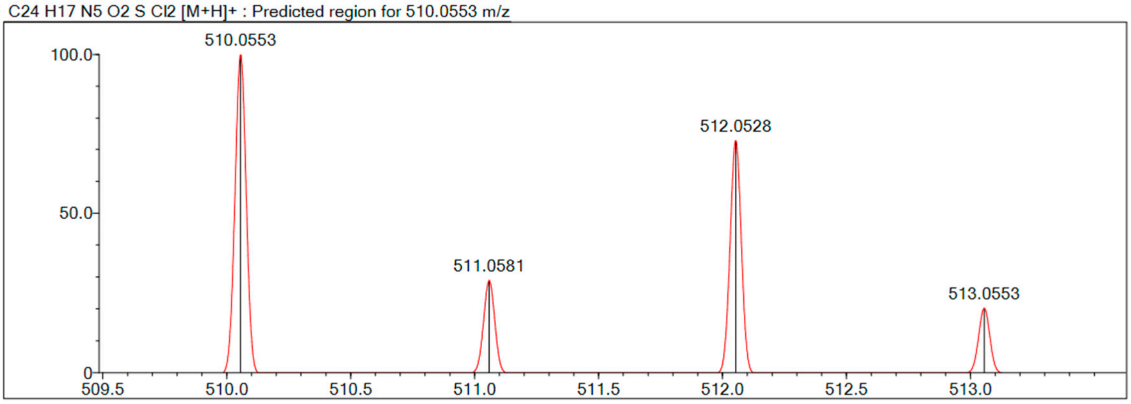

Rank	Score	Formula (M)	Ion	Meas. m/z	Pred. m/z	Df. (mDa)	Df. (ppm)	Iso	DBE
1	96.89	C24 H17 N5 O2 S Cl2	[M+H]+	510.0557	510.0553	0.4	0.78	96.89	18.0

Figure S10. Mass spectrum of compound **6d**

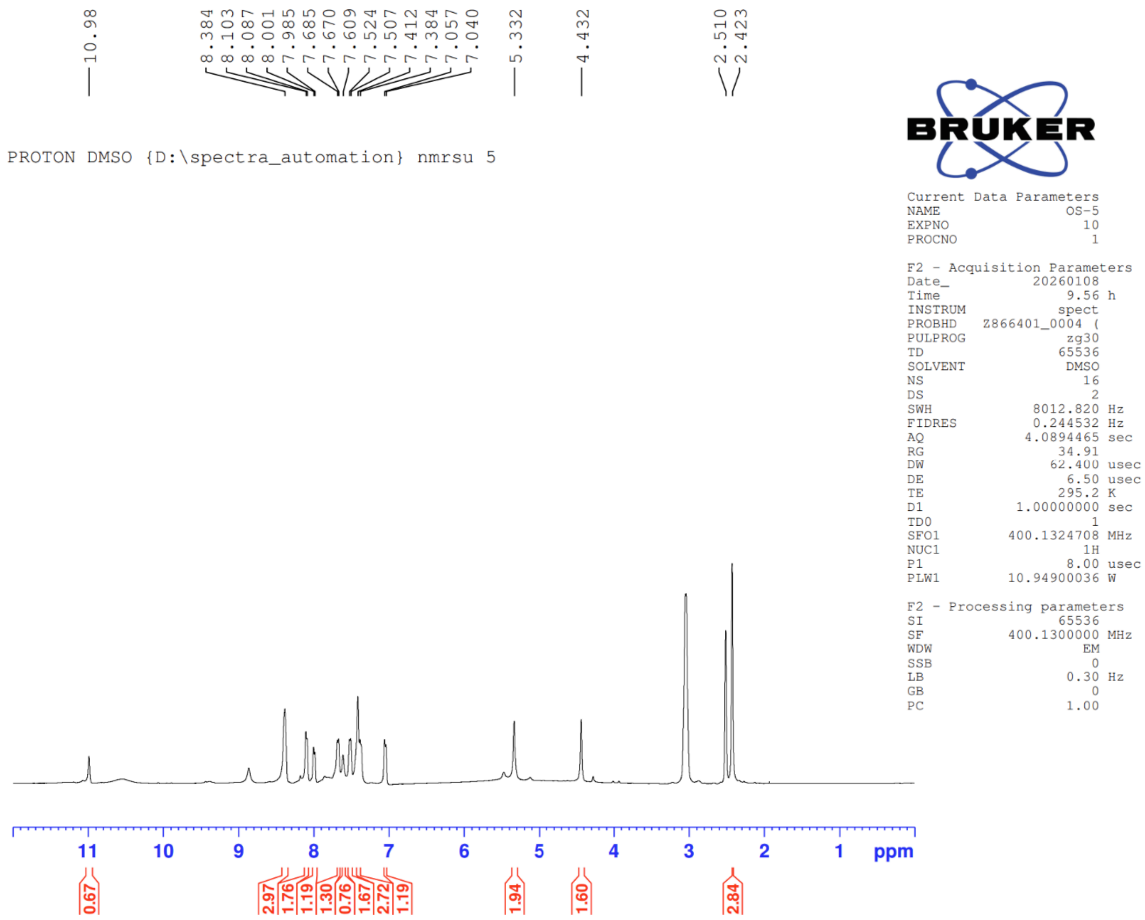

Figure S11. ^{1}H-NMR spectrum of compound **6e**

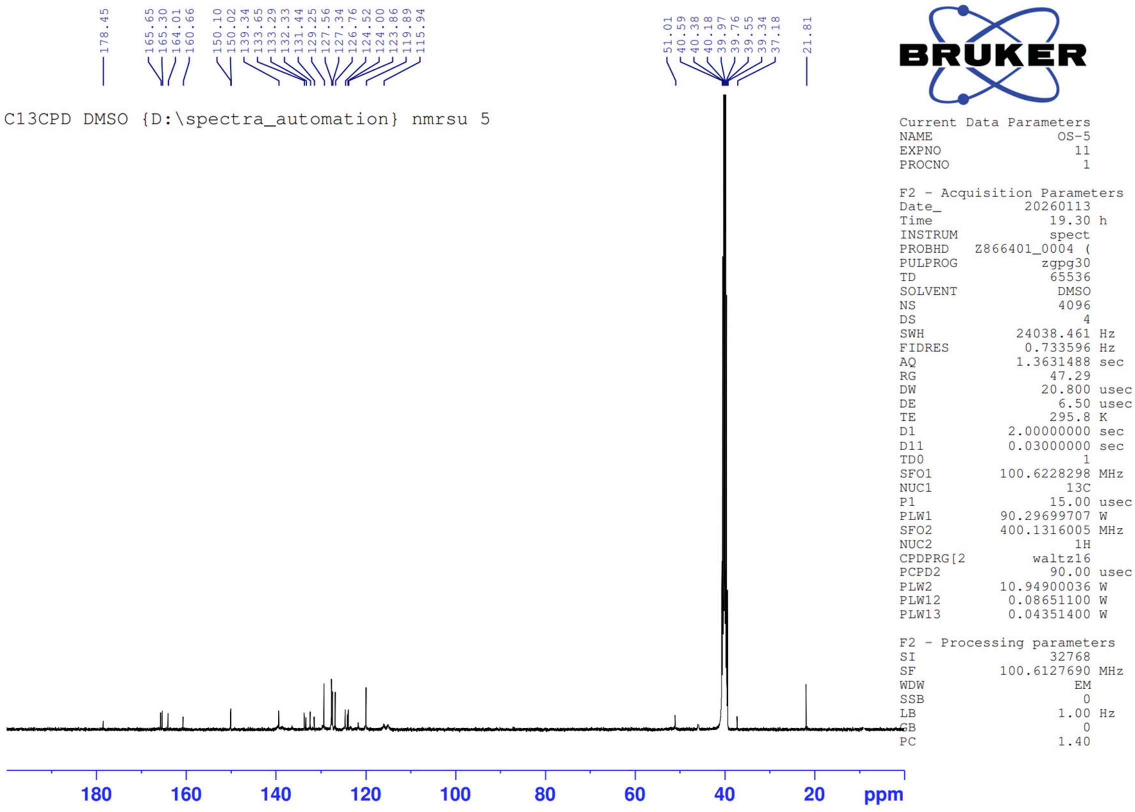

Figure S12. ^{13}C-NMR spectrum of compound **6e**

Data File: C:\LabSolutions\Data\Analiz\uac\OS-5_179.lcd

Elmt	Val.	Min	Max	Elmt	Val.	Min	Max	Elmt	Val.	Min	Max	Elmt	Val.	Min	Max	Use Adduct
H	1	0	25	O	2	0	4	Cl	1	0	0	I	3	0	0	H
B	3	0	0	F	1	0	0	Br	1	0	0					
C	4	0	35	P	3	0	0	Ru	2	0	0					
N	3	0	7	S	2	1	1	Pd	2	0	0					

Error Margin (ppm): 5
HC Ratio: unlimited
Max Isotopes: 3
MSn Iso RI (%): 10.00

DBE Range: 5.0 - 25.0
Apply N Rule: yes
Isotope RI (%): 1.00
MSn Logic Mode: AND

Electron Ions: both
Use MSn Info: yes
Isotope Res: 9000
Max Results: 50

Event#: 1 MS(E+)   Ret. Time : 1.760 -> 2.453   Scan# : 265 -> 369

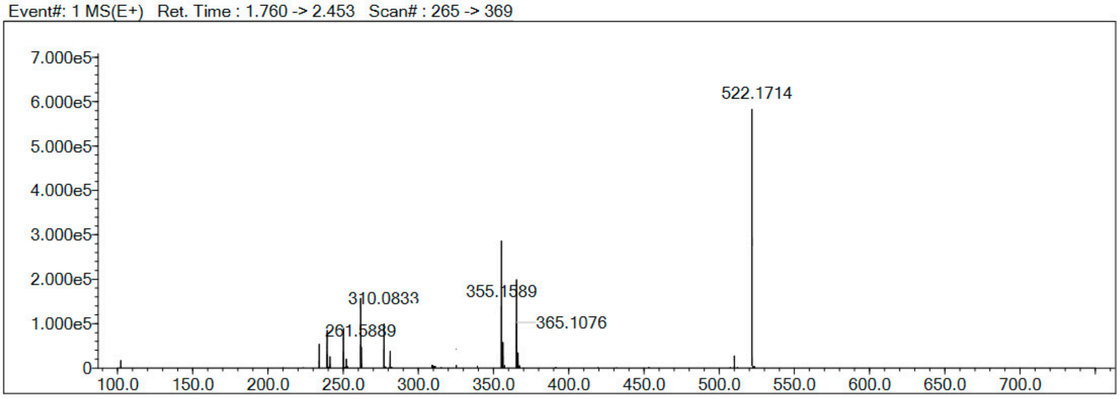

Measured region for 522.1714 m/z

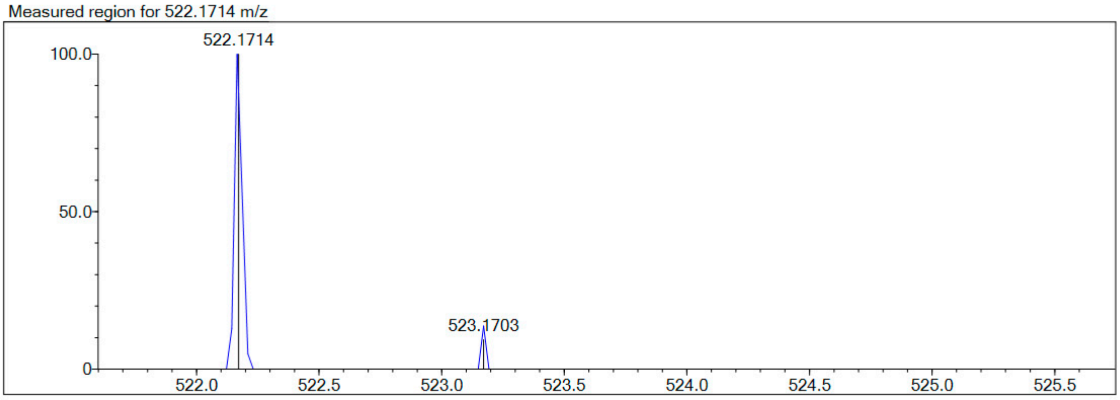

C28 H23 N7 O2 S [M+H]+ : Predicted region for 522.1707 m/z

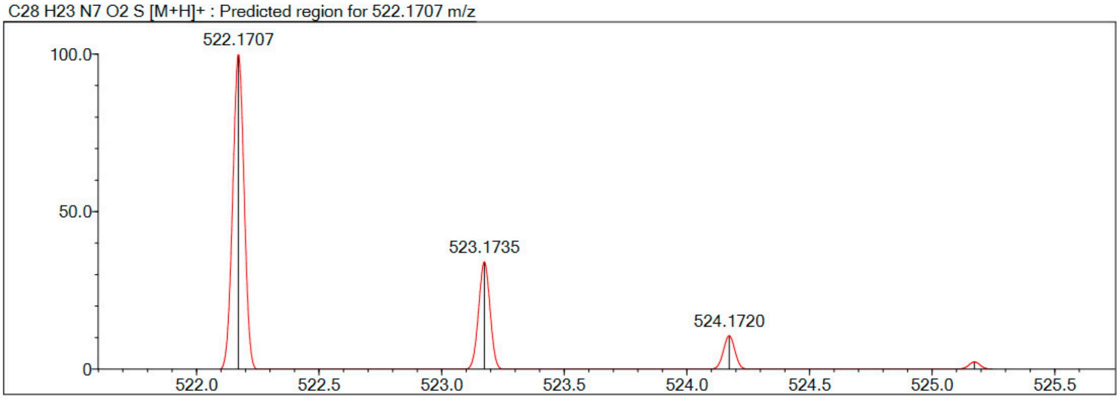

Rank	Score	Formula (M)	Ion	Meas. m/z	Pred. m/z	Df. (mDa)	Df. (ppm)	Iso	DBE
1	0.00	C28 H23 N7 O2 S	[M+H]+	522.1714	522.1707	0.7	1.34	0.00	21.0

Figure S13. Mass spectrum of compound **6e**

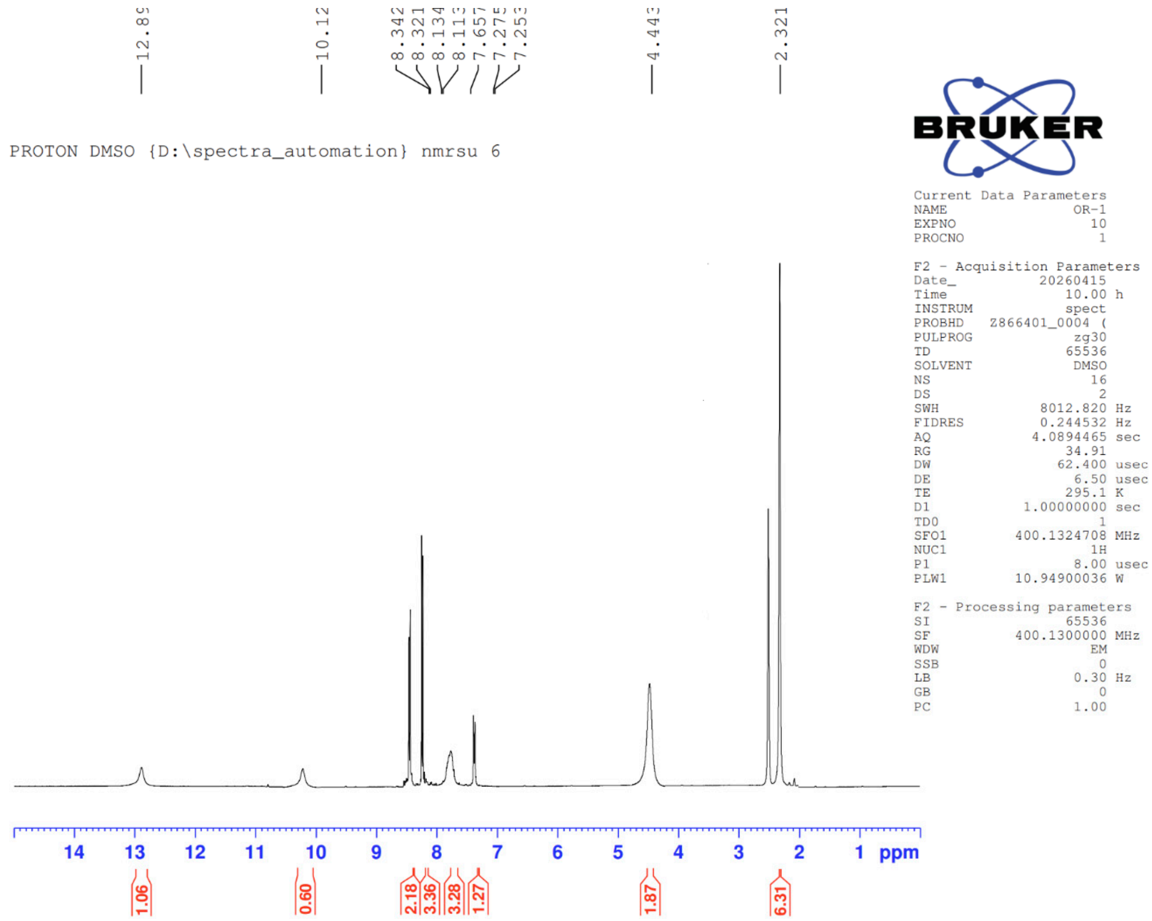

Figure S14. ^{1}H-NMR spectrum of compound **7a**

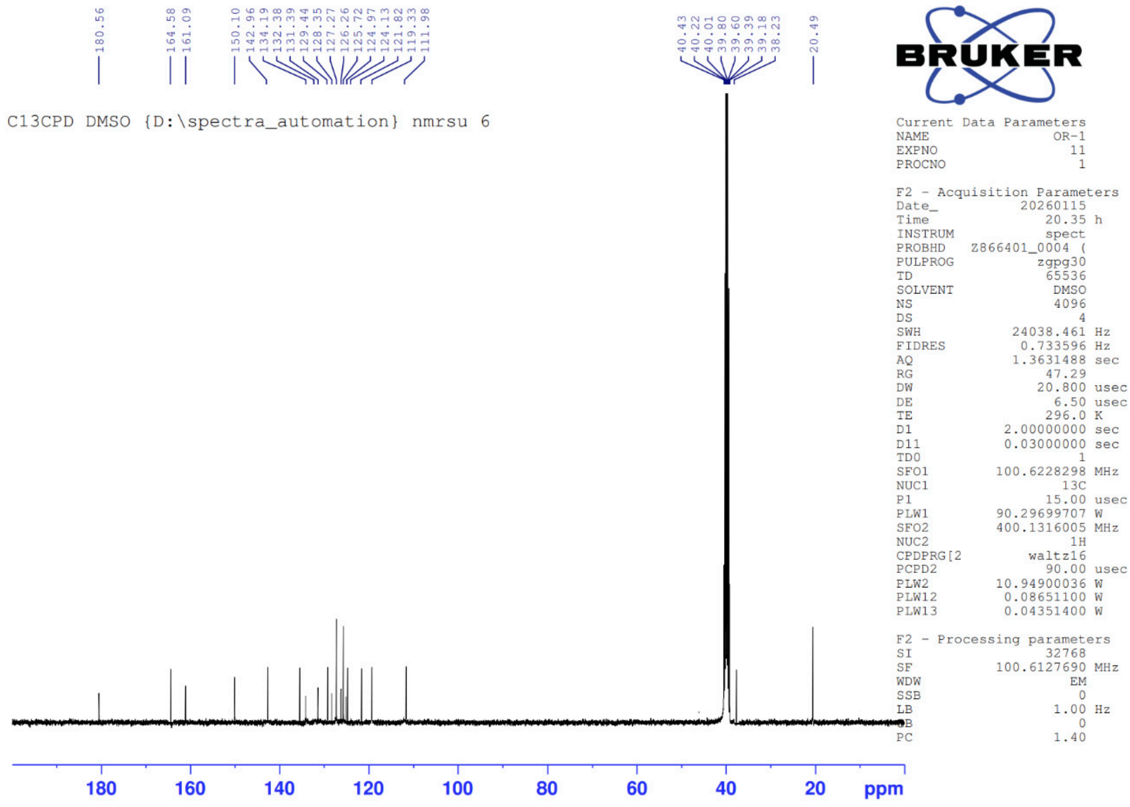

Figure S15. ^{13}C-NMR spectrum of compound **7a**

Data File: C:\LabSolutions\Data\Analiz\uac\OR-1_153.lcd

Elmt	Val.	Min	Max	Elmt	Val.	Min	Max	Elmt	Val.	Min	Max	Elmt	Val.	Min	Max	Use Adduct
H	1	0	25	O	2	0	5	Cl	1	2	2	I	3	0	0	H
B	3	0	0	F	1	0	0	Br	1	0	0					
C	4	0	30	P	3	0	0	Ru	2	0	0					
N	3	0	7	S	2	1	1	Pd	2	0	0					

Error Margin (ppm): 5
HC Ratio: unlimited
Max Isotopes: 3
MSn Iso RI (%): 10.00

DBE Range: 5.0 - 30.0
Apply N Rule: yes
Isotope RI (%): 1.00
MSn Logic Mode: AND

Electron Ions: both
Use MSn Info: yes
Isotope Res: 9000
Max Results: 50

Event#: 1 MS(E+) Ret. Time : 3.333 Scan# : 501

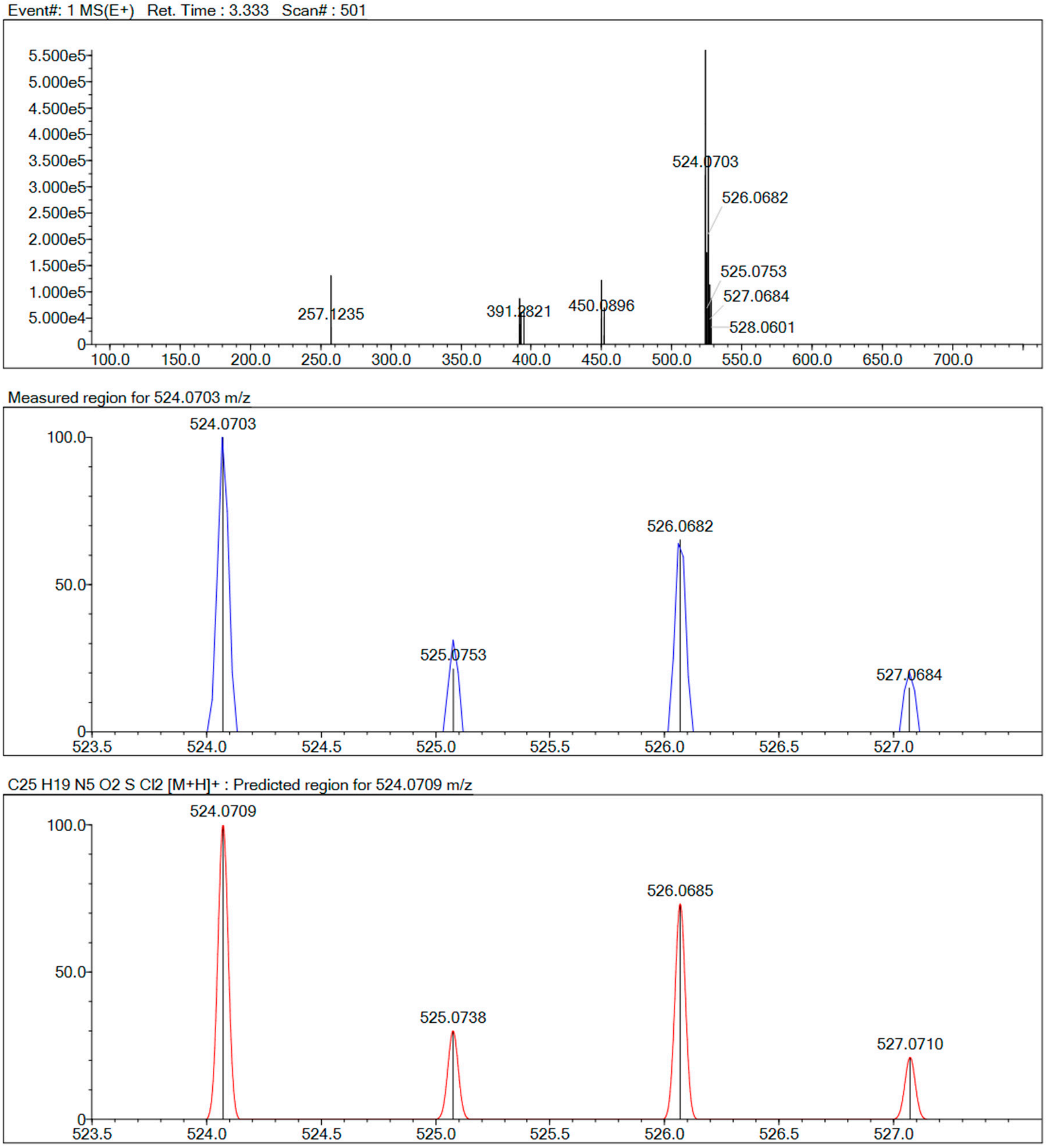

Rank	Score	Formula (M)	Ion	Meas. m/z	Pred. m/z	Df. (mDa)	Df. (ppm)	Iso	DBE
1	76.15	C25 H19 N5 O2 S Cl2	[M+H]+	524.0703	524.0709	-0.6	-1.14	76.42	18.0

Figure S16. Mass spectrum of compound **7a**

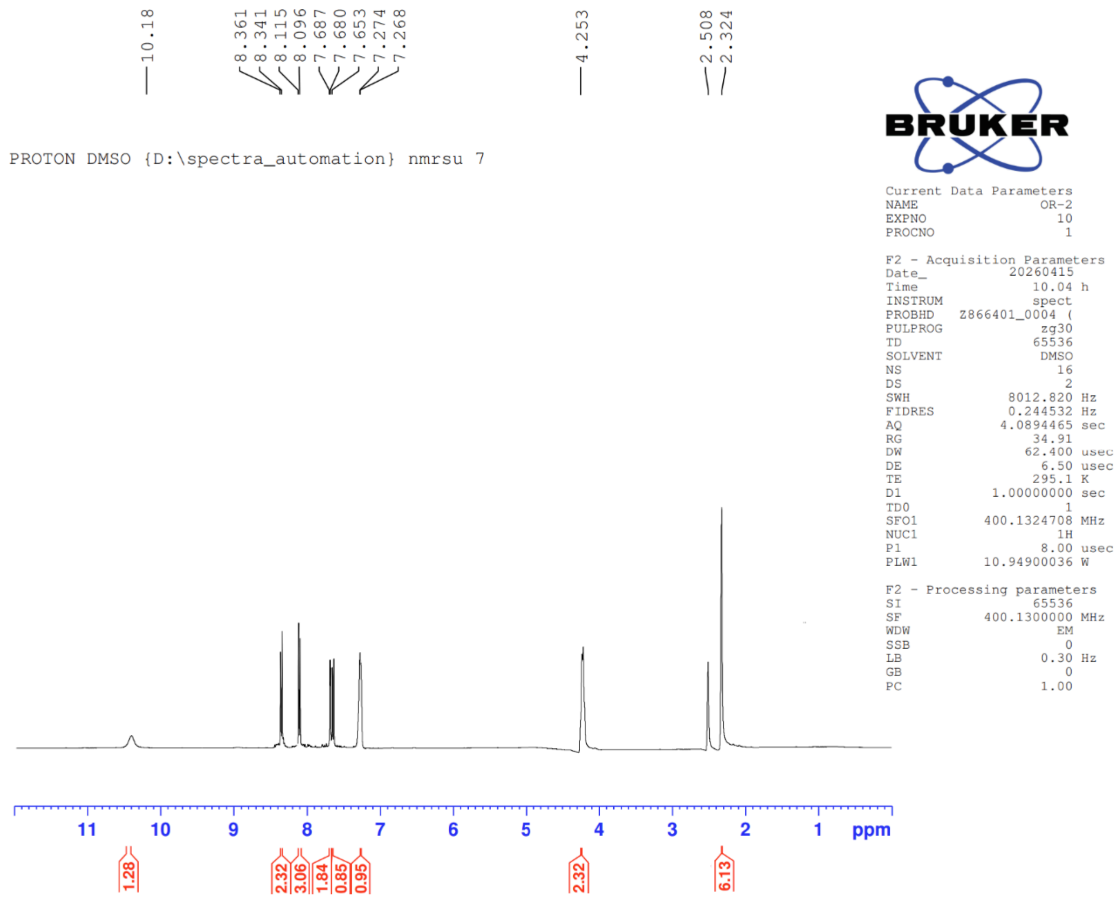

Figure S17. ^{1}H-NMR spectrum of compound **7b**

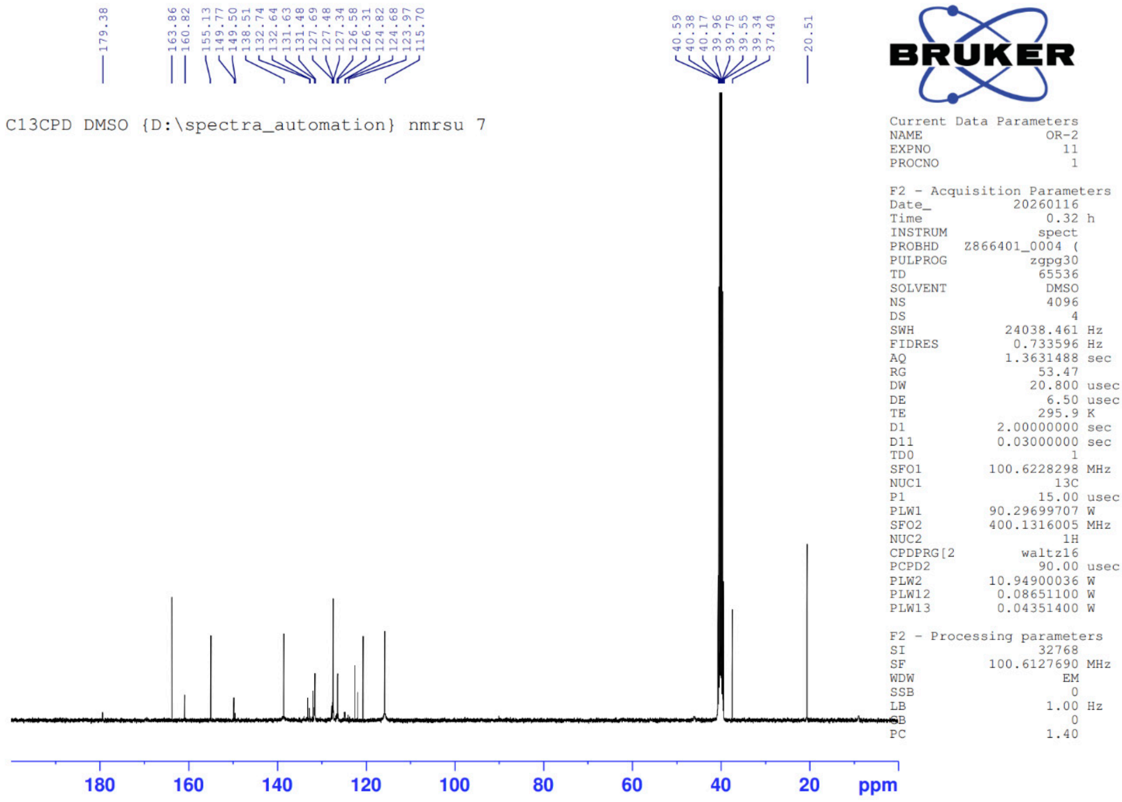

Figure S18. ^{13}C-NMR spectrum of compound **7b**

Data File: C:\LabSolutions\Data\Analiz\uac\OR-2_154.lcd

Elmt	Val.	Min	Max	Elmt	Val.	Min	Max	Elmt	Val.	Min	Max	Elmt	Val.	Min	Max	Use Adduct
H	1	0	25	O	2	0	5	Cl	1	2	2	I	3	0	0	H
B	3	0	0	F	1	0	0	Br	1	0	0					
C	4	0	30	P	3	0	0	Ru	2	0	0					
N	3	0	7	S	2	1	1	Pd	2	0	0					

Error Margin (ppm): 5
HC Ratio: unlimited
Max Isotopes: 3
MSn Iso RI (%): 10.00

DBE Range: 5.0 - 30.0
Apply N Rule: yes
Isotope RI (%): 1.00
MSn Logic Mode: AND

Electron Ions: both
Use MSn Info: yes
Isotope Res: 9000
Max Results: 50

Event#: 1 MS(E+) Ret. Time : 2.853 Scan# : 429

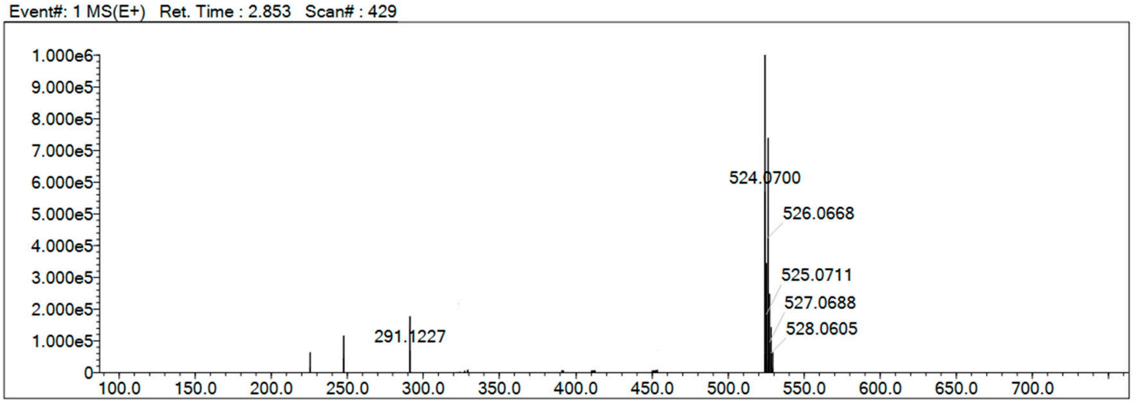

Measured region for 524.0700 m/z

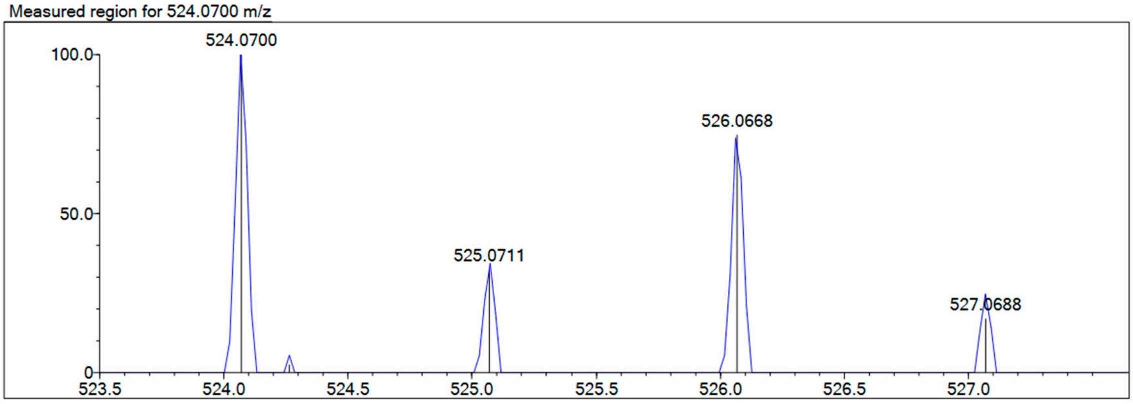

C25 H19 N5 O2 S Cl2 [M+H]+ : Predicted region for 524.0709 m/z

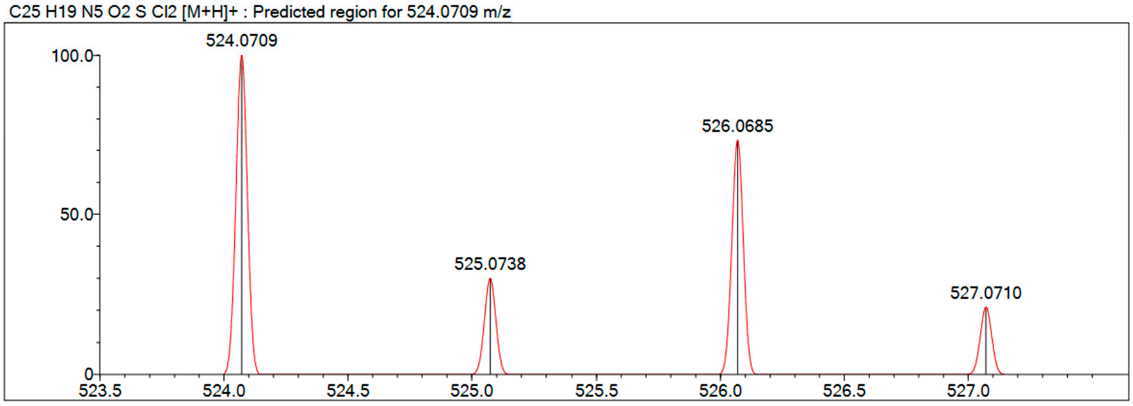

Rank	Score	Formula (M)	Ion	Meas. m/z	Pred. m/z	Df. (mDa)	Df. (ppm)	Iso	DBE
1	81.19	C25 H19 N5 O2 S Cl2	[M+H]+	524.0700	524.0709	-0.9	-1.72	82.67	18.0

Figure S19. Mass spectrum of compound **7b**

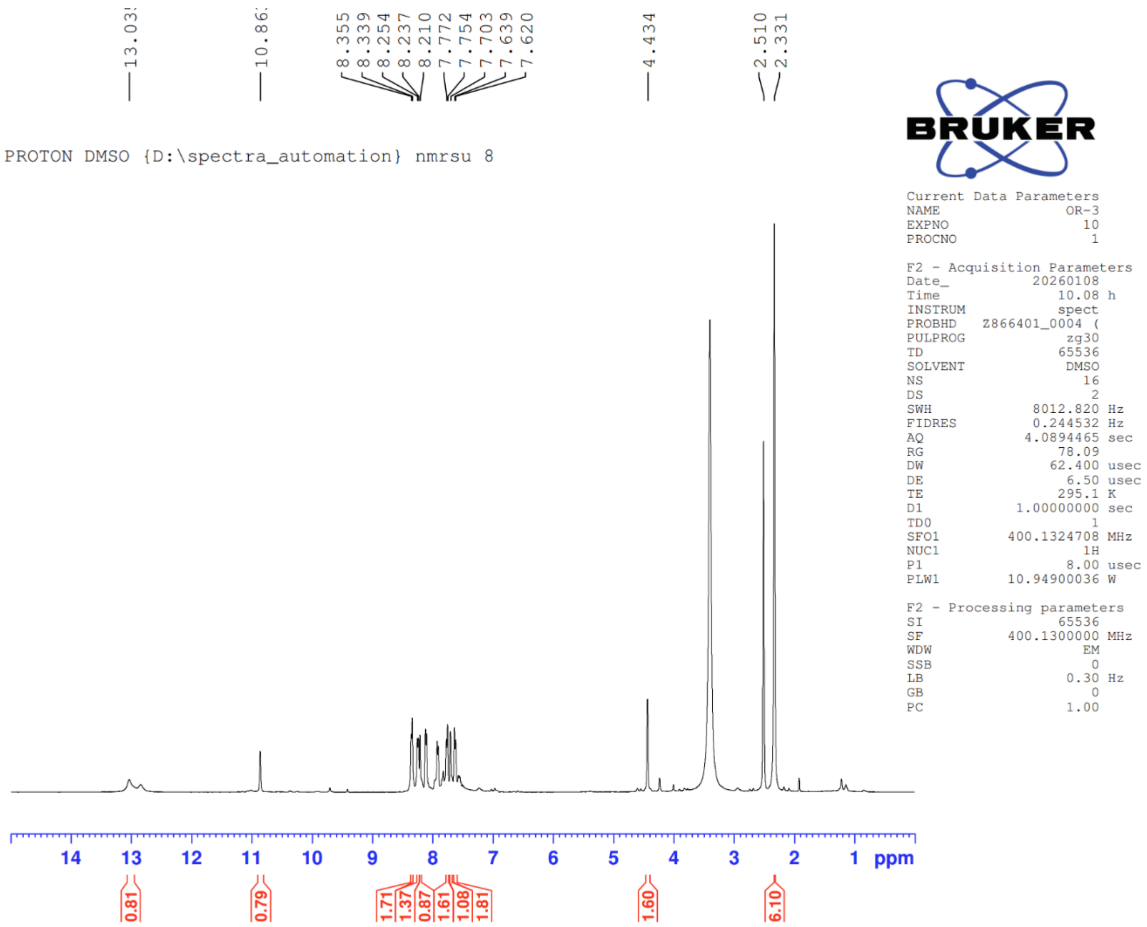

Figure S20. ^{1}H-NMR spectrum of compound **7c**

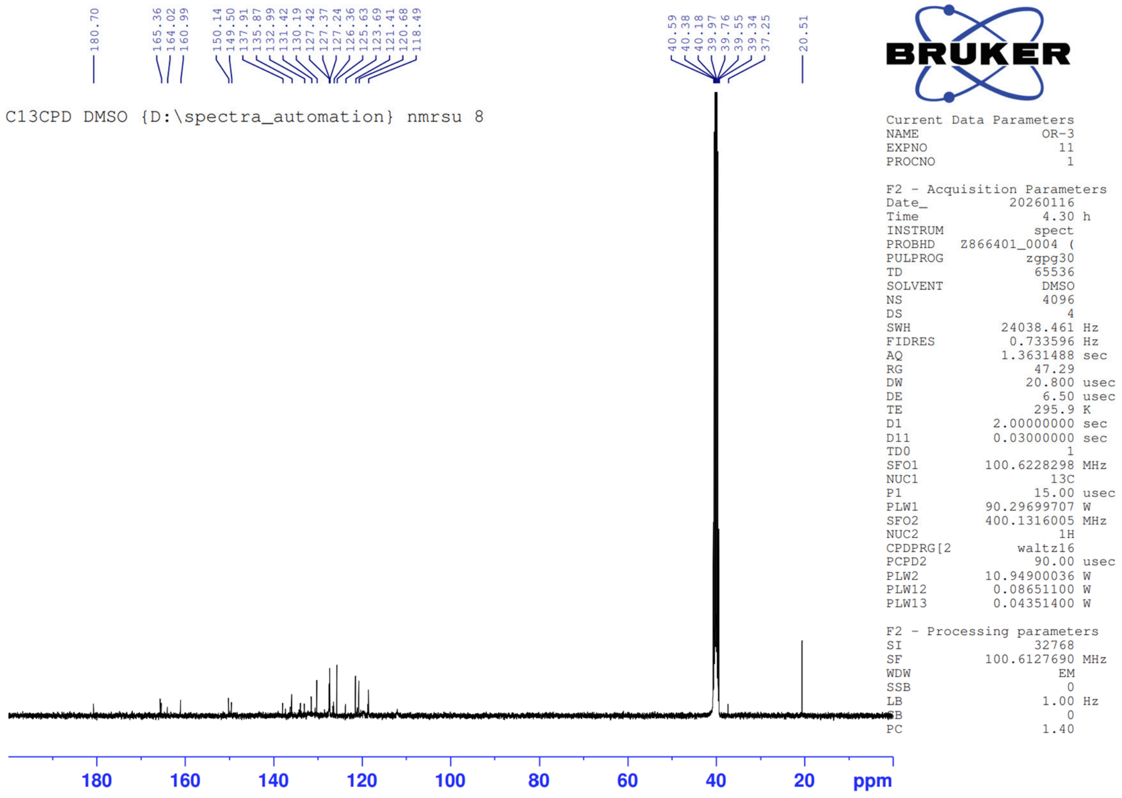

Figure S21. ^{13}C-NMR spectrum of compound **7c**

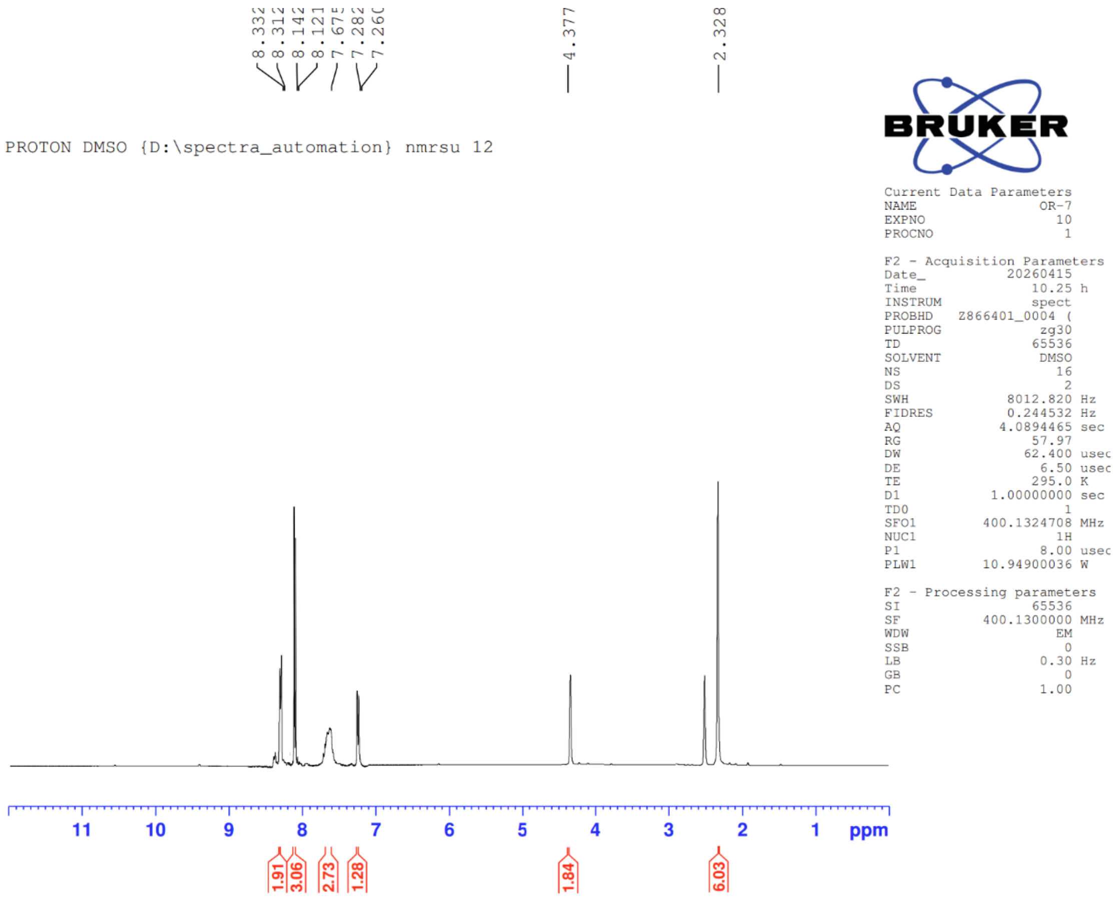

Figure S22. ^{1}H-NMR spectrum of compound **7d**

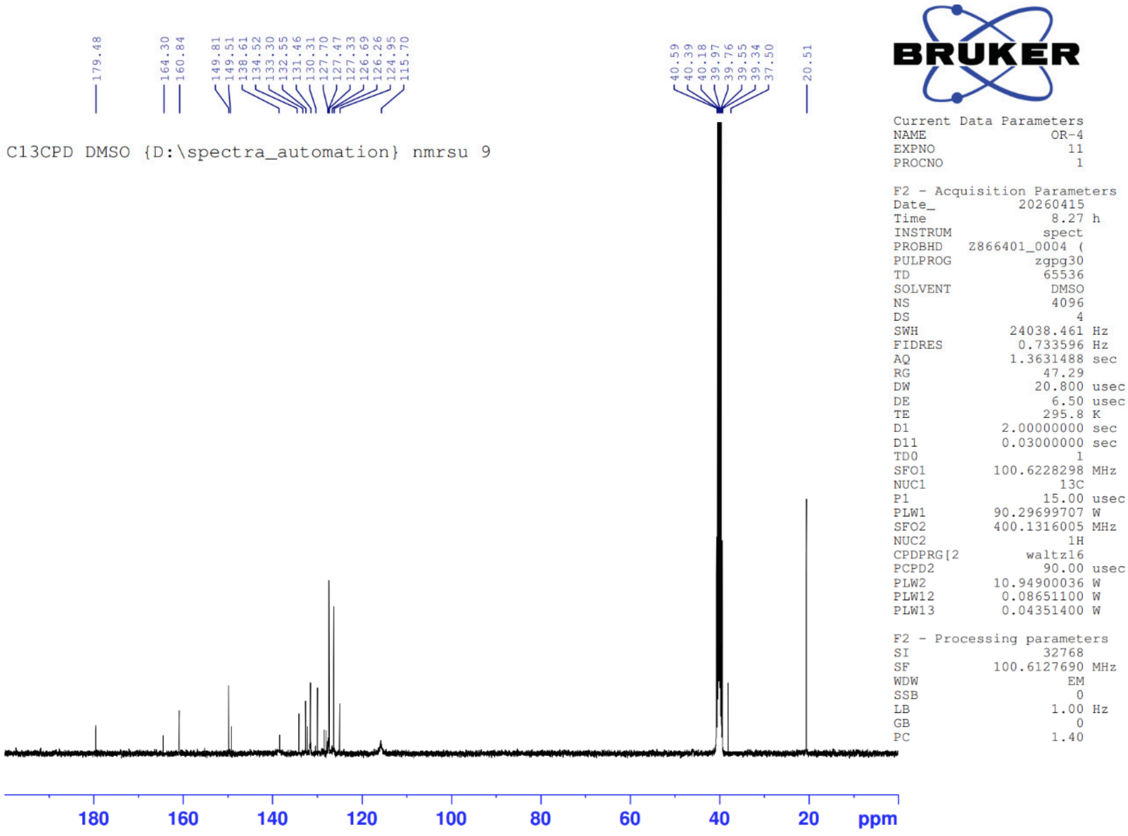

Figure S23. ^{13}C-NMR spectrum of compound **7d**

Data File: C:\LabSolutions\Data\Analiz\uac\OR-4_156.lcd

Elmt	Val.	Min	Max	Elmt	Val.	Min	Max	Elmt	Val.	Min	Max	Elmt	Val.	Min	Max	Use Adduct
H	1	0	25	O	2	0	5	Cl	1	2	2	I	3	0	0	H
B	3	0	0	F	1	0	0	Br	1	0	0					
C	4	0	30	P	3	0	0	Ru	2	0	0					
N	3	0	7	S	2	1	1	Pd	2	0	0					

Error Margin (ppm): 5
HC Ratio: unlimited
Max Isotopes: 3
MSn Iso RI (%): 10.00

DBE Range: 5.0 - 30.0
Apply N Rule: yes
Isotope RI (%): 1.00
MSn Logic Mode: AND

Electron Ions: both
Use MSn Info: yes
Isotope Res: 9000
Max Results: 50

Event#: 1 MS(E+) Ret. Time : 2.267 Scan# : 341

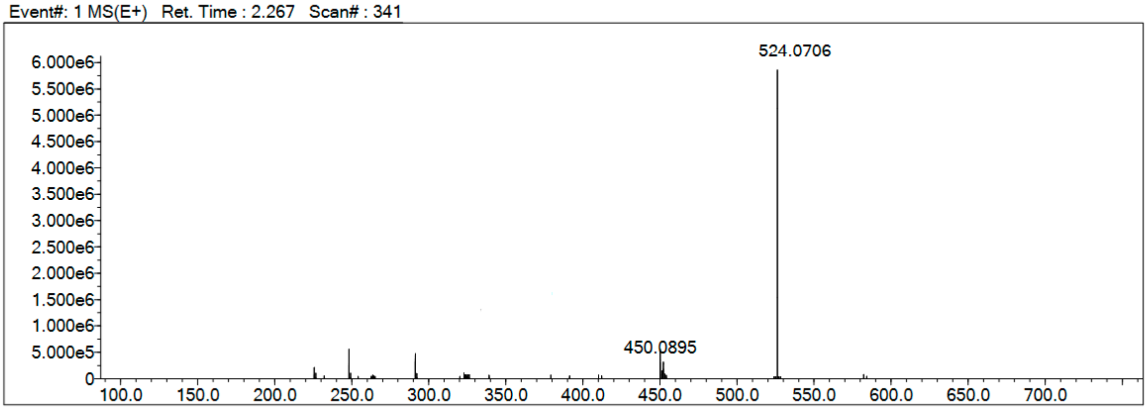

Measured region for 524.0706 m/z

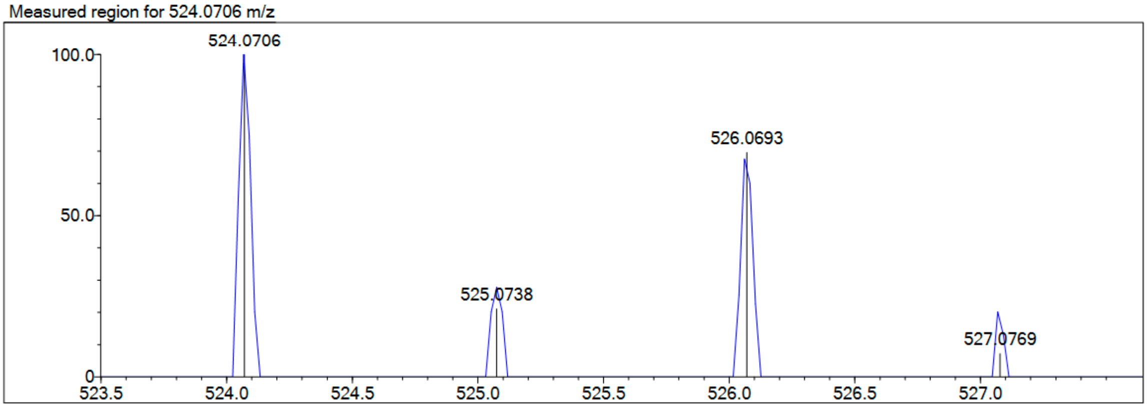

C25 H19 N5 O2 S Cl2 [M+H]+ : Predicted region for 524.0709 m/z

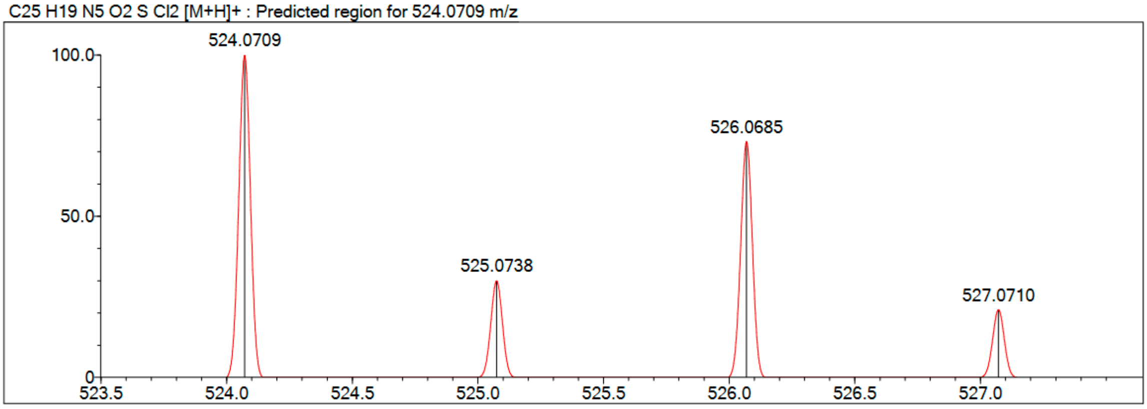

Rank	Score	Formula (M)	Ion	Meas. m/z	Pred. m/z	Df. (mDa)	Df. (ppm)	Iso	DBE
1	77.45	C25 H19 N5 O2 S Cl2	[M+H]+	524.0706	524.0709	-0.3	-0.57	77.45	18.0

Figure S24. Mass spectrum of compound **7d**

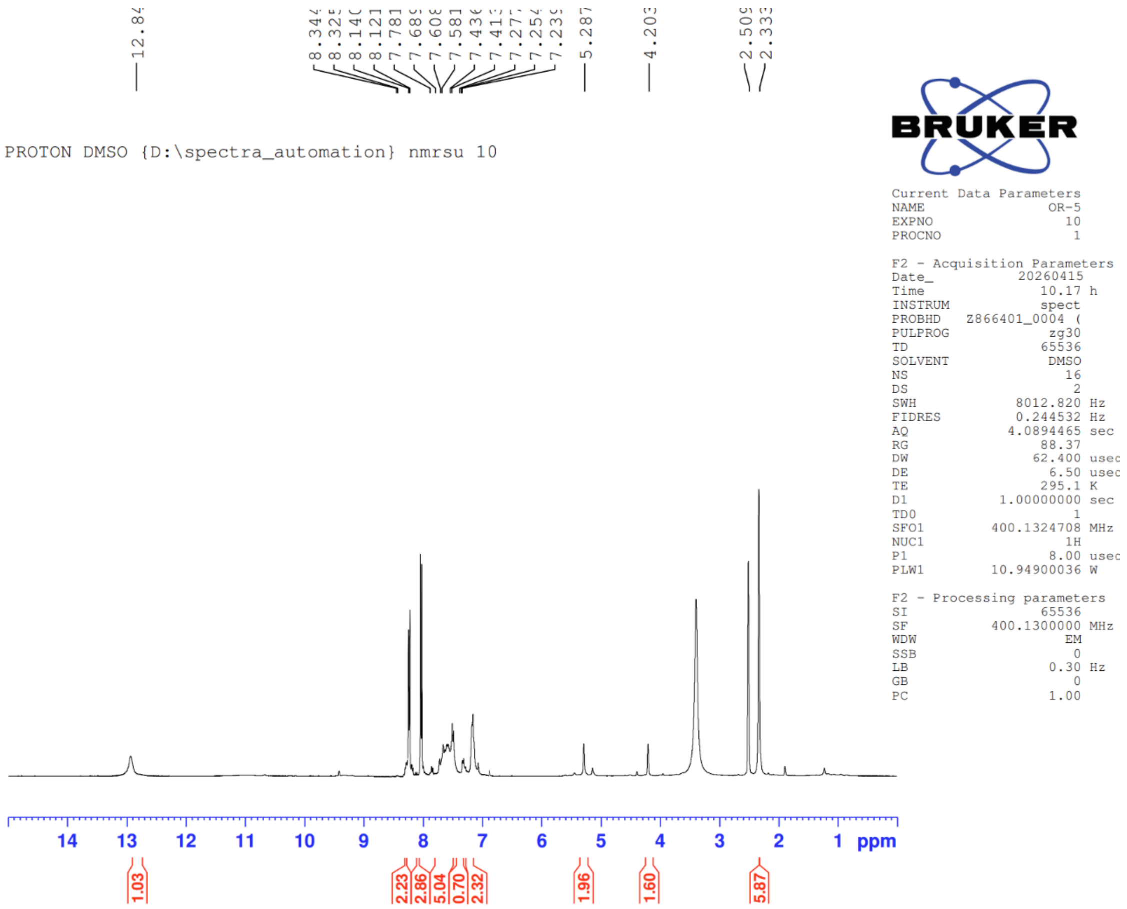

Figure S25. ^{1}H-NMR spectrum of compound **7e**

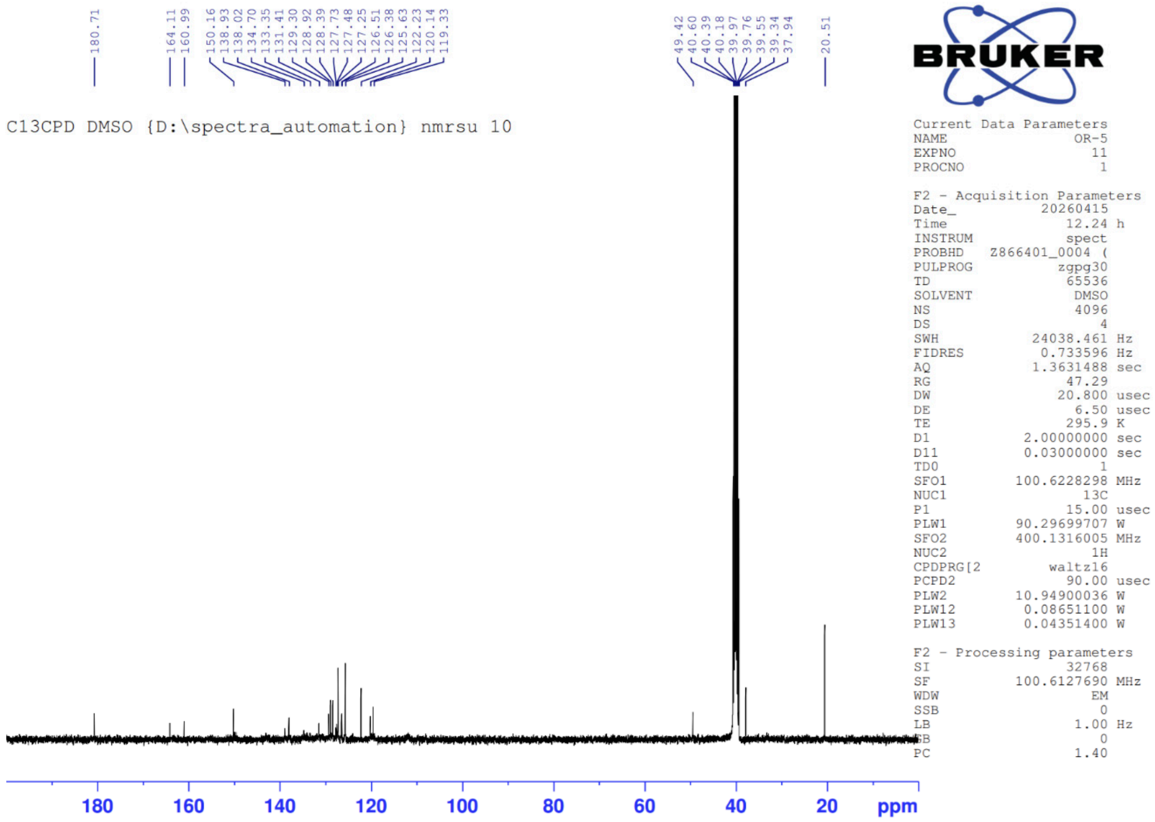

Figure S26. ^{13}C-NMR spectrum of compound **7e**

Data File: C:\LabSolutions\Data\Analiz\uac\OR-5_157.lcd

Elmt	Val.	Min	Max	Elmt	Val.	Min	Max	Elmt	Val.	Min	Max	Elmt	Val.	Min	Max	Use Adduct
H	1	0	25	O	2	0	5	Cl	1	0	0	I	3	0	0	H
B	3	0	0	F	1	0	0	Br	1	0	0					
C	4	0	30	P	3	0	0	Ru	2	0	0					
N	3	0	7	S	2	1	1	Pd	2	0	0					

Error Margin (ppm):	5	DBE Range:	5.0 - 30.0	Electron Ions:	both
HC Ratio:	unlimited	Apply N Rule:	yes	Use MSn Info:	yes
Max Isotopes:	3	Isotope RI (%):	1.00	Isotope Res:	9000
MSn Iso RI (%):	10.00	MSn Logic Mode:	AND	Max Results:	50

Event#: 1 MS(E+) Ret. Time : 2.227 Scan# : 335

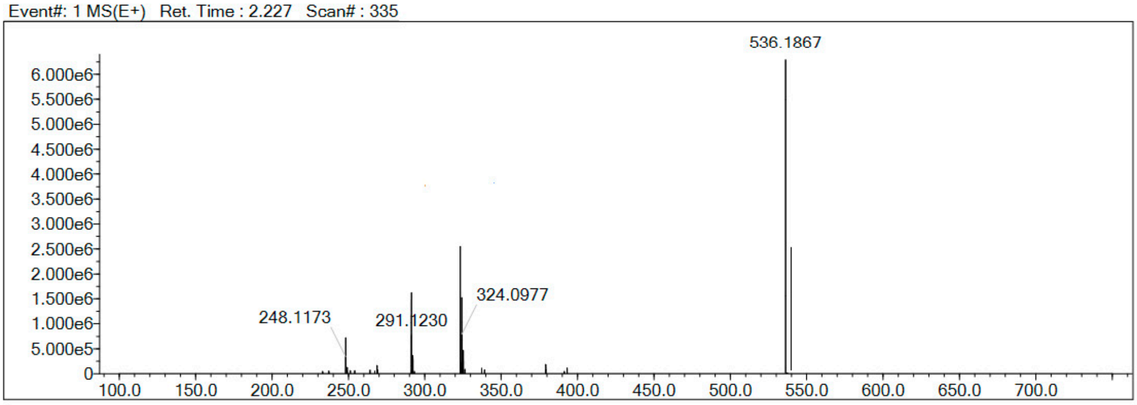

Measured region for 536.1867 m/z

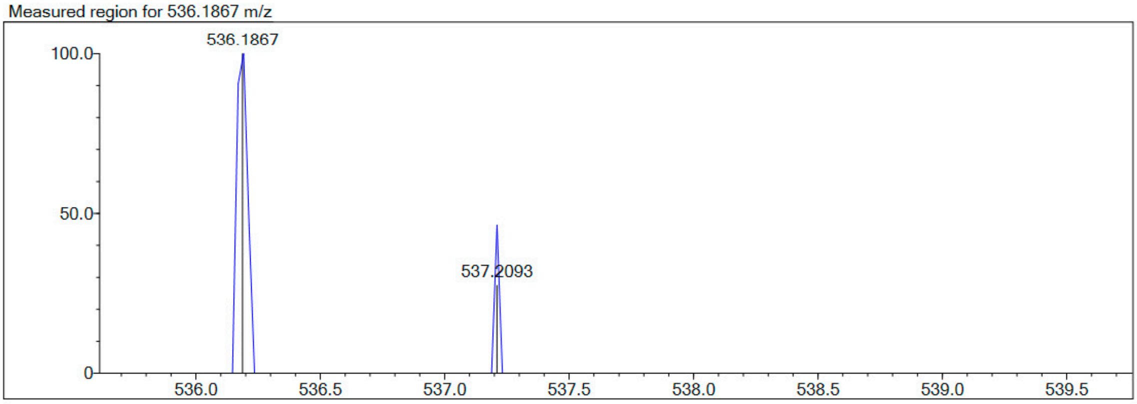

C29 H25 N7 O2 S [M+H]+ : Predicted region for 536.1863 m/z

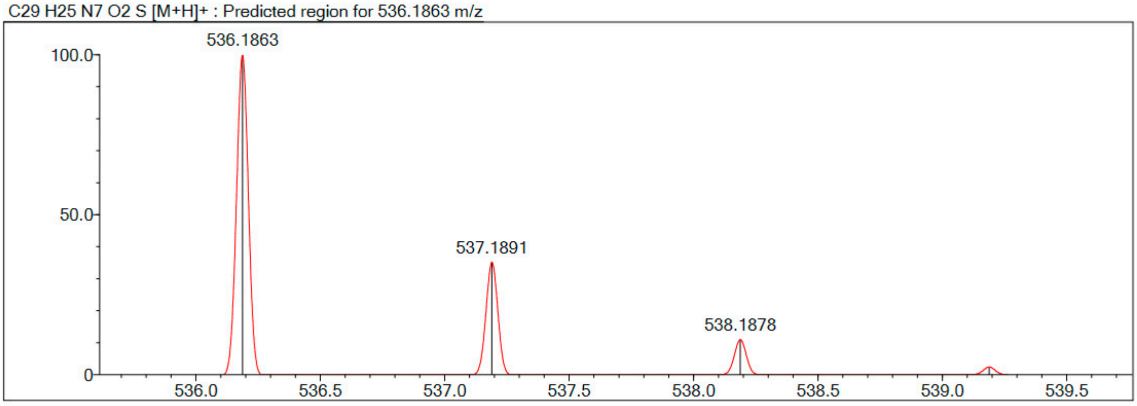

Rank	Score	Formula (M)	Ion	Meas. m/z	Pred. m/z	Df. (mDa)	Df. (ppm)	Iso	DBE
1	0.00	C29 H25 N7 O2 S	[M+H]+	536.1867	536.1863	0.4	0.75	0.00	21.0

Figure S27. Mass spectrum of compound **7e**
